# Chemotaxonomy of Mycotoxigenic Small-Spored *Alternaria* Fungi – Do Multitoxin Mixtures Act as an Indicator for Species Differentiation?

**DOI:** 10.3389/fmicb.2018.01368

**Published:** 2018-07-03

**Authors:** Theresa Zwickel, Sandra M. Kahl, Michael Rychlik, Marina E. H. Müller

**Affiliations:** ^1^Federal Institute for Risk Assessment (BfR), Berlin, Germany; ^2^Chair of Analytical Food Chemistry, Technical University of Munich, Munich, Germany; ^3^Leibniz Centre for Agricultural Landscape Research (ZALF), Müncheberg, Germany; ^4^Institute of Biochemistry and Biology, University of Potsdam, Potsdam, Germany

**Keywords:** small-spored *Alternaria* fungi, *Alternaria* species-groups, *Alternaria* mycotoxins, chemotaxonomy, secondary metabolite profiling, LC-MS/MS, wheat, perylene quinone derivatives

## Abstract

Necrotrophic as well as saprophytic small-spored *Alternaria (A.)* species are annually responsible for major losses of agricultural products, such as cereal crops, associated with the contamination of food and feedstuff with potential health-endangering *Alternaria* toxins. Knowledge of the metabolic capabilities of different species-groups to form mycotoxins is of importance for a reliable risk assessment. 93 *Alternaria* strains belonging to the four species groups *Alternaria tenuissima*, *A. arborescens*, *A. alternata*, and *A. infectoria* were isolated from winter wheat kernels harvested from fields in Germany and Russia and incubated under equal conditions. Chemical analysis by means of an HPLC-MS/MS multi-*Alternaria*-toxin-method showed that 95% of all strains were able to form at least one of the targeted 17 non-host specific *Alternaria* toxins. Simultaneous production of up to 15 (modified) *Alternaria* toxins by members of the *A. tenuissima*, *A. arborescens*, *A. alternata* species-groups and up to seven toxins by *A. infectoria* strains was demonstrated. Overall tenuazonic acid was the most extensively formed mycotoxin followed by alternariol and alternariol mono methylether, whereas altertoxin I was the most frequently detected toxin. Sulfoconjugated modifications of alternariol, alternariol mono methylether, altenuisol and altenuene were frequently determined. Unknown perylene quinone derivatives were additionally detected. Strains of the species-group *A. infectoria* could be segregated from strains of the other three species-groups due to significantly lower toxin levels and the specific production of infectopyrone. Apart from infectopyrone, alterperylenol was also frequently produced by 95% of the *A. infectoria* strains. Neither by the concentration nor by the composition of the targeted *Alternaria* toxins a differentiation between the species-groups *A. alternata*, *A. tenuissima* and *A. arborescens* was possible.

## Introduction

The fungal genus *Alternaria* NEES is, on a global scale, one of the most common molds, growing in different environments under various conditions and on numerous substrates. Currently, approximately 300 species are described and, based on molecular sequencing, divided into 26 sections (Lawrence et al., 2013; Woudenberg et al., 2013, 2015; Lee et al., 2015). *Alternaria alternata, A. tenuissima, A. arborescens*, and *A. infectoria* are representatives of the most prominent ones. Interestingly, some members of the *A.* *infectoria* species-group have, among the other strictly asexual species-groups, the unique characteristic of producing a sexual state (*Lewia* spec.) (Woudenberg et al., 2013; Lawrence et al., 2014). Identification and classification of small-spored *Alternaria* spp. by means of individual approaches often seem remarkably difficult. The use of polyphasic approaches, under the aspects of morphological, molecular and chemotaxonomic characteristics, appears to provide a more successful classification tool within the genus *Alternaria* (Kahl et al., 2015; da Cruz Cabral et al., 2017). Especially closely related species, commonly found on cereals, like members of the *A. alternata, A. arborescens.*, and *A. tenuissima* species-groups are difficult to distinguish, whereas the successful separation of *A. infectoria* was shown in previous studies (Andersen et al., 2002, 2009; Kahl et al., 2015; Zwickel et al., 2016a).

*Alternaria* species can exhibit different lifestyles and modes of nutrition such as saprophytic, endophytic or pathogenic (Thomma, 2003; Lawrence et al., 2014). Saprophytic *Alternaria* strains, also known as decomposers, feed off dead matter and are often responsible for postharvest spoilage of agricultural commodities and spoilage of opened stored foodstuff in households. Besides that, they are important members of the rhizosphere microbiome and crucial for plant health (Ostry, 2008; Logrieco et al., 2009). Necrotrophic *Alternaria* pathogens infest a significant number of living host plants and cause several plant diseases such as early blight in tomato and potato, stem canker in tomato, leaf blight in carrot and black point disease in wheat kernels. These pathogens destroy the plant cells in the process of colonization by producing cell wall-degrading enzymes and secondary metabolites, which may lead to major harvest losses (Hasan, 1999; Thomma, 2003; Mercado Vergnes et al., 2006; Logrieco et al., 2009).

**FIGURE 1:**
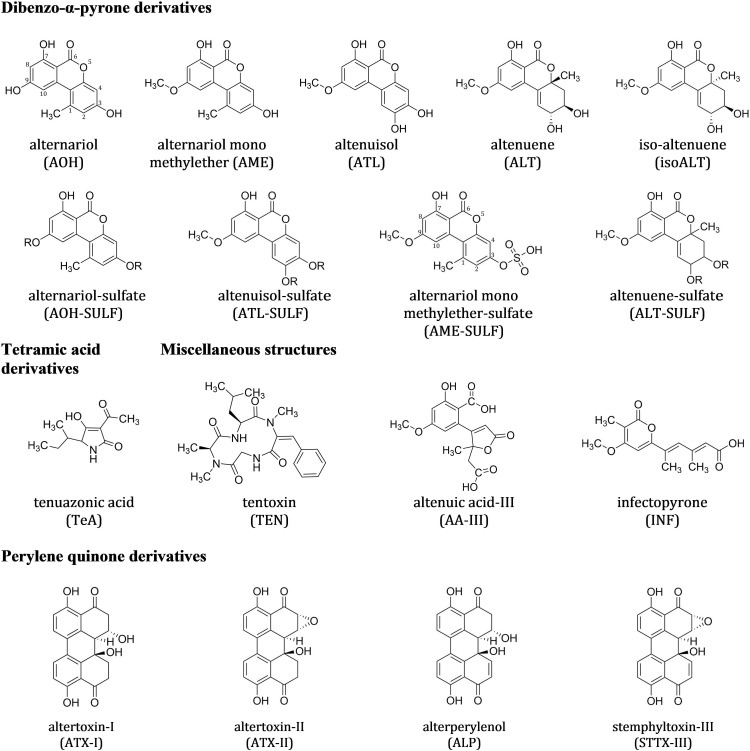
Chemical structures of the 17 non-host specific *Alternaria* toxins (ATs) analyzed in this study. Altenuene and iso-altenuene were analyzed as sum of both analytes [sum(iso)ALT]. “R” marks the possible positions of the sulfate group.

**FIGURE 2:**
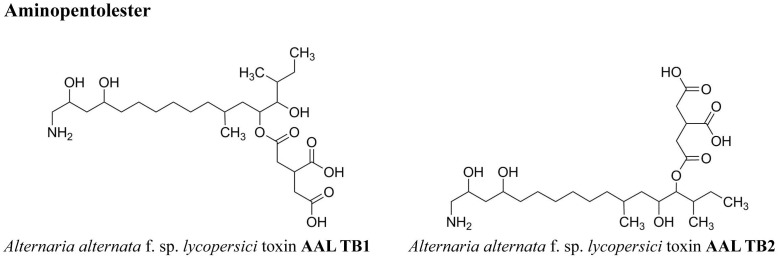
Chemical structures of the host specific *Alternaria* toxins (ATs) analyzed in this study.

Currently, at least 70 secondary metabolites, highly varying in their chemical structures and behavior, are known to be formed by different *Alternaria* species. Some are phytotoxins, associated with the infection, colonization and death of plants (Rotem, 1994; Tsuge et al., 2013). Others are potent mycotoxins with the potential of having adverse effects on human and animal health. These *Alternaria* toxins (ATs) can be divided in host specific and non-host specific toxins (Meena et al., 2017) or grouped according to their chemical structures (EFSA, 2011), which is the preferred approach in food safety issues. The dibenzo-α-pyrone derivatives group contains alternariol (AOH) and several modified forms of it such as alternariol mono methylether (AME), altenuene (ALT), isoaltenuene (isoALT) and altenuisol (ATL) (Nemecek et al., 2012). Next come the tetramic acid derivatives group with tenuazonic acid (TeA), followed by the perylene quinone derivatives [altertoxin I, II, III (ATX-I, -II, -III), alterperylenol also named alteichin (ALP) and stemphyltoxin III (STTX-III)]. The fourth group contains miscellaneous structures such as tentoxin (TEN), altenuic acid III (AA-III) and infectopyrone (INF), which was first isolated from *A. infectoria* in 2003 and can also be formed by *Stemphylium* and *Ulocladium* species (Ostenfeld Larsen et al., 2003; Andersen and Hollensted, 2008; Nemecek et al., 2013; Zwickel et al., 2016b). The toxins in these four groups belong to the non-host specific toxins and can be formed by various *Alternaria* strains on a broad spectrum of living host plants and agricultural products, such as harvested wheat kernels, cereal products, vegetables, fruits, and oil seeds (Ostry, 2008; Logrieco et al., 2009; EFSA, 2011; Müller and Korn, 2013; Lee et al., 2015; Fraeyman et al., 2017). As opposed to the previous groups, the group of the aminopentol esters contains representatives for host specific toxins, the AAL toxins (e.g., AAL TB1, and TB2), named by their host specific fungus *A. alternata* f. sp. *lycopersici.* AAL toxins are described as molecular determinants of the *Alternaria* stem canker disease of tomatoes (Winter et al., 1996; Yamagishi et al., 2006). Structures of *Alternaria* toxins investigated in this study are summarized in **Figures 1, 2**. Biotransformation of ATs by plants or the fungus itself results in modified forms of the original toxin conjugated with, e.g., glucose or activated sulfuric acid (Levsen et al., 2005; Rychlik et al., 2014). Sulfated forms of AOH, AME, and ATL were demonstrated to be formed by two *A. tenuissima* isolates in rice and wheat (Zwickel et al., 2016a). AOH and AME sulfates were also detected in tomato products (Walravens et al., 2016). Formation of beta-D-glucopyranosides of AOH and D-glucopyranosides of AME was demonstrated in plant cells, more precisely suspension cultures of tobacco BY-2 cells (Hildebrand et al., 2015).

A matter of concern regarding human and animal health is the toxicity of certain ATs. Even though a recent *in vivo* study (Schuchardt et al., 2014) reported that no toxic or genotoxic effect of AOH in bone marrow was revealed in the micronucleus assay and no systemic genotoxicity was indicated in the comet assay with liver tissue, AOH and AME are still classified as genotoxic substances by EFSA (EFSA Arcella et al., 2016). In contrast to the *in vivo* results several reviewed *in vitro* studies give evidence for the genotoxicity of AOH by generating single- and double-strand breaks due to the formation of reactive oxygen species and interaction with the DNA topoisomerase (Solhaug et al., 2016). Even though ATX-I-III and STTX-III are all identified as potent mutagens (Stack and Prival, 1986; Stack and Mazzola, 1989) only ATX-II and STTX-III show a higher genotoxic potential than AOH in V79 cells and ATX-II in human cells compared to AOH and AME (Schwarz et al., 2012; Fleck et al., 2016). Moreover, ATX-II has a 50-fold, and STTX-III a 40-fold higher mutagenic potency than AOH (Fleck et al., 2012, 2016). ATX-II shows the highest toxic potential against HeLa cells, followed by AOH, ATL, AME, ATX-I, ALT, and TeA (Pero et al., 1973). Although TeA shows a low toxicity in *in vitro* studies, it was classified as possibly harmful due to its acute oral toxicity in mice and rats (EFSA, 2011). It causes severe *in vivo* effects in rats, mice, dogs and monkeys ranging from emesis, salivation, tachycardia, hemorrhages, and hemorrhagic gastro-enteropathy (Ostry, 2008; Lee et al., 2015; Fraeyman et al., 2017).

As a consequence of the ubiquitous distribution of *Alternaria* species, co-occurrence of multicomponent *Alternaria* toxin mixtures in food and feed is constantly reported (EFSA, 2011; Patriarca, 2016; Zwickel et al., 2016b; Escriva et al., 2017). Due to the aforementioned toxic effects of single ATs an exposure to multiple mycotoxins is likely to create an even stronger adverse effect on human and animal health. For a reliable risk assessment, it is crucial to know to what extent different *Alternaria* species can form ATs. Moreover, secondary metabolite profiles can provide helpful information for the differentiation of *Alternaria* species in a polyphasic approach as well as supporting a more practicable risk assessment by knowing the frequency of species’ occurrence with potential toxin formation. In a previous study we investigated the influence of the extrinsic factors, temperature, substrate and incubation time on the quantitative AT production of three *Alternaria* isolates by means of an HPLC-MS/MS multi-toxin method (Zwickel et al., 2016a). Based on these results we extended the approach to take a closer look at the metabolic capability of 93 *Alternaria* strains isolated from wheat in Germany and Russia belonging to the four species-groups *A.* *tenuissima, A.* *arborescens, A. alternata*, and *A.* *infectoria* to form ATs under equal *in vitro* conditions. A detailed overview is given of the quantitative production of AOH, AME, ALT, isoALT, ATL, TeA, TEN, ATX-I, ATX-II, STTX-III, ALP, AA-III, and AAL TB1/TB2. Furthermore, screening for modified ATs, such as sulfates of AOH, AME, ATL, and ALT and the toxin infectopyrone was carried out.

## Materials and Methods

### Chemicals, Reagents and Standard Solutions

Analytical grade water (0.055 μS/cm) was generated from a Milli-Q system for all experiments (Merck, Darmstadt, Germany). LiChrosolv LC-MS hypergrade quality methanol and acetonitrile, HPLC grade ammonium acetate, ammonium hydroxide solution (25%) and acetic acid (100%) were purchased from Merck, (Darmstadt, Germany). Analytical standards (>99% purity) of AOH, AME, TEN, TeA and a mixture of AAL-TB1 and TB2 toxin were purchased from Sigma-Aldrich (St. Louis, MO, United States). ATX-I, ATX-II, and STTX-III were received via preparative HPLC of *Alternaria* isolates (Professor Metzler’s group at the Institute of Applied Bioscience at the Karlsruhe Institute of Technology, Karlsruhe, Germany) (Fleck et al., 2014). ALT, iso(ALT), AA-III, and ATL were synthesized (Professor Podlech’s group at the Institute of Organic Chemistry at the Karlsruhe Institute of Technology, Karlsruhe, Germany) (Nemecek et al., 2012, 2013). ALP was received via preparative HPLC of *Alternaria* isolates. Single toxin stock solutions were prepared in methanol in concentrations between 100 μg/mL and 1 mg/mL depending on the remaining amount of toxin. Stock solutions were kept at −30°C. The actual concentration of each stock solution was obtained by means of ultraviolet spectroscopy (UV-1700 Pharma Spec, Shimadzu, Kyoto, Japan) as described by Zwickel et al. (2016b). Mixed working solutions (1 μg/mL) of each toxin were prepared by diluting the stock solution with a mixture of 75% 1 mM ammonium acetate (pH 9) and 25% methanol. Calibration mix solutions were prepared freshly each day in a range from 1 to 1000 ng/mL for the quantification of the ATs by means of an external calibration using weighted linear regression (weighing factor 1/y).

### Biological Material

A total of 93 *Alternaria* fungal isolates were used in this study belonging to four different species-groups and originating from three habitats; 21 from the *A. alternata*, 35 from the *A. tenuissima*, 26 from the *A. infectoria* and 11 from the *A. arborescens* species-group. 89 strains were isolated from kernel samples collected from two winter wheat fields in the Uckermark region in Germany (31 isolates from Helpt and 34 isolates from Steinfurth) and from a field in the region of Novosibirsk in Russia (24 isolates). Classification of the isolates was carried out in a previous study by means of a polyphasic approach based on morphological analysis (macromorphology of the colonies and three-dimensional sporulation patterns), molecular analysis using TEF1-α gene fragment sequence and mycotoxin analysis of TeA, AOH, AME, ALT, and ATX-I (Kahl et al., 2015). *Alternaria* was isolated from grains after incubation on potato dextrose agar (PDA, Carl Roth, Karlsruhe, Germany). Single spore stock cultures of all fungal isolates are stored on sterile wheat kernels at −20°C. They can be found in the cultures collection of the Leibniz-Centre of Agricultural Landscape Research (ZALF) Müncheberg, Germany. Isolates’ names consist of a code of country (first capital letter; G: Germany, R: Russia), region (capital letter; H: Helpt, N: Novosibirsk, St: Steinfurth), identification number and sporulation group (a: *A. alternata*, ab: *A. arborescens*, i: *A. infectoria* and t: *A. tenuissima*). Four additional strains were obtained from the personal collection of Dr. E. G. Simmons (EGS) and from the Centraalbureau voor Schimmelcultures, Fungal Biodiversity Centre (Utrecht) (CSB) (Supplementary Table S1).

### *In Vitro* Conditions and Extraction of *Alternaria* Toxins

For each of the 93 isolates a spore suspension with 1 × 10^5^ spores/mL was produced after incubation on potato-carrot-agar described by Kahl et al. (2015). For each isolate five centrifuge tubes (15 mL) were filled with 0.2 g of rice and 250 μL of demineralized water (*n* = 465). Tubes were sterilized twice before inoculation. The rice samples were inoculated with 50 μL of the respective spore suspension and incubated at 25°C for 14 days. These were the optimized conditions from a previous study under which different *Alternaria* isolates formed all the investigated *Alternaria* toxins in considerable amounts to compare the different species-groups (Zwickel et al., 2016a). 25°C was chosen as the incubation temperature to simulate natural field conditions during the ripening stage of wheat ears. Five control rice samples were not inoculated but incubated under the same conditions to prove the absence of contamination. After 14 days of incubation samples were frozen at −20°C to stop the fungal growth and mycotoxin production. After defrosting samples were treated as described by Zwickel et al. (2016a).

### ESI-HPLC-MS/MS Analysis

High Performance Liquid Chromatography (HPLC) analyses were performed on a 1100 HPLC system from Agilent Technologies (Santa Clara, CA, United States) coupled to an API 4000 (SCIEX, Foster City, CA, United States) triple quadrupole mass spectrometer. The system was equipped with an electrospray interface (ESI) (Turbo V, SCIEX, Foster City, CA, United States) and negative and positive ionization was used during acquisition. Multi-*Alternaria*-toxin separation was performed on a reversed phase Gemini NX-C18 HPLC column (5 μm particle size, 100 mm × 2.1 mm) equipped with a C18 precolumn cartridge system (0.3 mm) from Phenomenex (Aschaffenburg, Germany). Column oven temperature was set at 40°C and the autosampler was operated at 10°C. Binary gradient elution with a constant flow rate of 0.3 mL/min was used starting with 100% aqueous ammonium acetate solution [1 mM; pH adjusted to 9 with ammonium hydroxide solution (25%)] as solvent A and 0% methanol as solvent B. After 5 min of equilibration time 5 μl of sample solution were injected and solvent B was held at 0% for 1 min. At 1.2 min solvent B was set at 95% and was held for 5 min before the gradient was returned to starting conditions after 1 min. During sample measurement the mass spectrometer was operated in the negative electrospray ionization mode. The polarity was switched to positive mode for 3 min throughout equilibration time to avoid possible contamination with negatively loaded particles which can lead to suppression of the tenuazonic acid ions (Zwickel et al., 2016b). Multiple reaction monitoring (MRM) mode was used for targeted quantitative mycotoxin analysis. Two ion transitions for each target compound were selected and are summarized with the respective mass spectrometer and ion source parameters in Supplementary Table S2. Data acquisition and evaluation were performed with Analyst version 1.6.2 and MultiQuant version 3.0.2. (both from SCIEX, Foster City, CA, United States, 2013). The development, validation and applications of the HPLC-MS/MS method for food and *in vitro* samples have been precisely described (Zwickel et al., 2016a,b).

### Confirmation Criteria for LC-MS/MS Analysis

*Alternaria* toxins were considered to be positively identified when the following criteria from the Guidance document on identification of mycotoxins in food and feed (European Commission, 2017) were met. Firstly, as required for the chromatographic separation, a minimal acceptable retention time twice the retention time corresponding to the void volume of the column was achieved. The retention time of the respective toxin in the sample extract corresponded to that of the average of the calibrant standards measured in the same sequence within a tolerance of ±0.2 min. Secondly, as required for the triple quadrupole mass spectrometric detection, the ion ratio of the two selected ion transitions for each analyte in the sample solution was within ±30% of that obtained from the average of the calibration standards. Limits of detection (LODs) and limits of quantification (LOQs) were determined according to DIN EN standard 32645 in extracts from not inoculated but incubated samples for all quantified ATs. LODs ranged from 0.5 to 3.0 μg/kg and LOQ ranged from 1.8 to 9.5 μg/kg (Supplementary Table S3).

### Screening for (Modified) *Alternaria* Toxins Without Reference Standard

Identification of modified *Alternaria* toxins with sulfuric acid was carried out by means of precursor ion scans of *m/z* 80, neutral loss scans of 80 Da and collision induced mass spectra (MS2) in high resolution and tandem mass spectrometry as described in a previous study (Zwickel et al., 2016a). ALT sulfate was additionally detected (*m/z* 371-*m/z* 291; *m/z* 371-*m/z* 229). High-resolution mass spectrometry (HRMS) analyses on an Accela HPLC system coupled to an Exactive (orbitrap) HCD (higher energy collisional dissociation) system fitted with a HESI II (heated-electrospray ionization) source (Thermo Fisher Scientific Inc., Waltham, MA, United States) were applied to confirm the detected modified ATs (AOH-, AME, ATL, and ATL-sulf) and screening for other suspected ATs such as infectopyrone, 4Z-infectopyrone, novae-zelandin A and B and modified structures of ATX-I, -II, ALP, and STTX-III. Measured accurate masses were compared to the respective calculated exact masses and confirmed as positively detected within a required mass accuracy of ≤5 ppm for *m/z*≥200 (European Commission, 2017). Ion source and scan parameters were set as described by Zwickel et al. (2016a): sheath gas flow 20 psi, spray voltage 4 kV, capillary temperature 350°C, capillary voltage −60 V, tube lens voltage −120 V, skimmer voltage −25 V, heater temperature 350°C, scan range 150.00-700.00 *m/z*, resolution was set to ultra-high [140000 FWHM (m/z 200)]. Ultra-high purity nitrogen (99.999%) was used as gas. Due to the lack of certified analytical calibrants for the dibenzo-α-pyrone-sulfates no valid amounts of formed modified toxins can be given in this study. Modified toxins were rated as either detected or not detected depending on compliance with the confirmation criteria based on retention time (±0.2 min) and ion ratio within 30% across all samples. Nevertheless, for an approximate statement regarding the magnitude of biotransformed *Alternaria* toxins, the ratio of the sulfated-dibenzo-α-pyrone peak area and the respective dibenzo-α-pyrone peak area was formed. Because the peak area is a variable which underlies measurements related fluctuations and therefore cannot be used for comparison of many samples measured on several days, the value of the quotient was multiplied with the content in mg/kg of the related dibenzo-α-pyrone which was calculated using the preceding calculation curve. The hereby calculated content in mg/kg of the most frequent sulfoconjugated mycotoxin AOH-sulf and AME-sulf was used for comparison between modified toxins in different samples and does not represent a valid amount.

### Statistical Analysis

**FIGURE 3:**
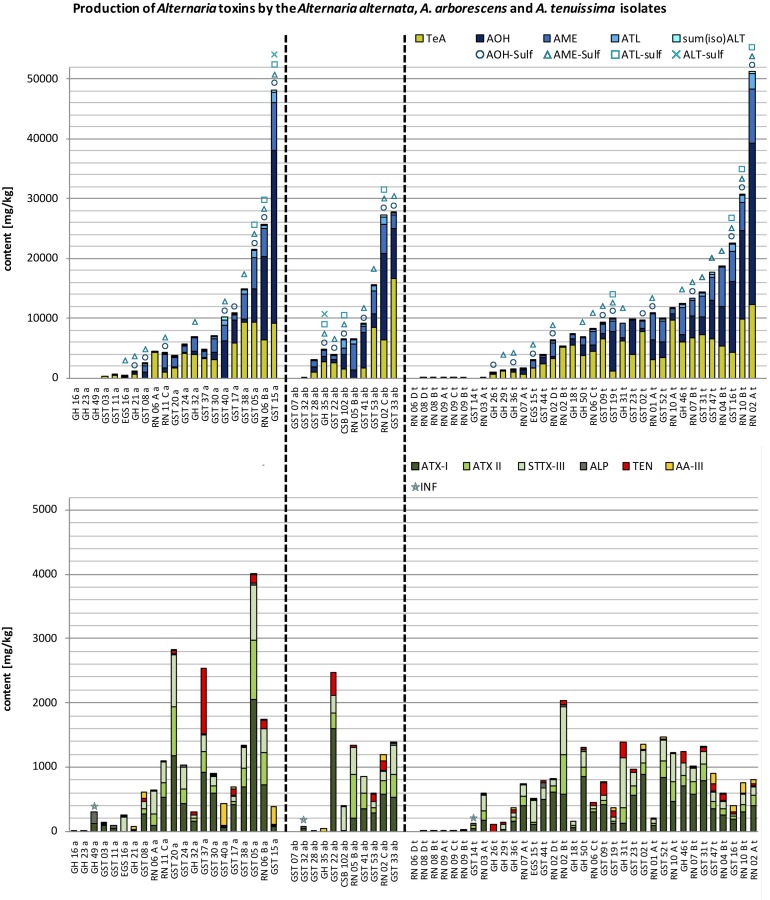
Stacked column charts – in ascending order regarding the total toxin production – of the quantitative production of tenuazonic acid (TeA), alternariol (AOH), alternariol mono methylether (AME), altenuisol (ATL) and the sum of altenuene and isoaltenuene [sum(iso)ALT] in comparison to the quantitative production of altertoxin I (ATX-I), altertoxin II (ATX-II), stemphyltoxin III (STTX-III), alterperylenol (ALP), tentoxin (TEN), and altenuic acid III (AA-III) after 14 days at 25°C in rice in mg/kg by the *A. alternata (a)*, *A. arborescens (ab)*, and *A. tenuissima (t)* species-group isolates (please note different scaling due to differences in concentration). The detection of alternariol sulfate (AOH-sulf), alternariol mono methylether sulfate (AME-sulf), altenuisol sulfate (ATL-sulf), altenuene sulfate (ALT-sulf) or infectopyrone (INF) is marked with the respective symbol.

**FIGURE 4:**
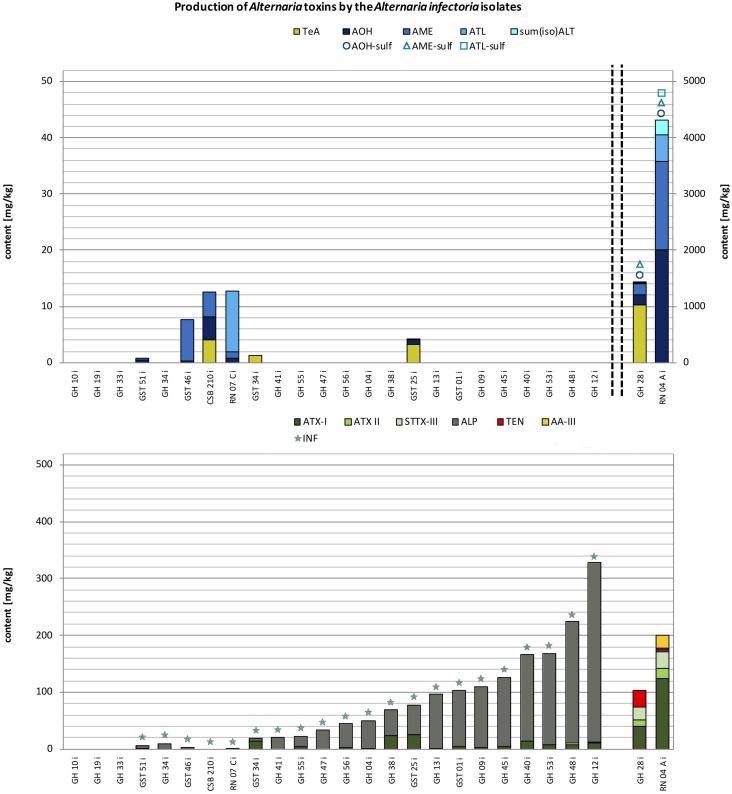
Stacked column charts – in ascending order regarding the total toxin production – of the quantitative production of tenuazonic acid (TeA), alternariol (AOH), alternariol mono methylether (AME), altenuisol (ATL) and the sum of altenuene and isoaltenuene [sum(iso)ALT] in comparison to the quantitative production of altertoxin I (ATX-I), altertoxin II (ATX-II), stemphyltoxin III (STTX-III), alterperylenol (ALP), tentoxin (TEN), and altenuic acid III (AA-III) after 14 days at 25°C in rice in mg/kg by the *A. infectoria* species-group isolates (please note different scaling due to differences in concentration). The detection of alternariol sulfate (AOH-sulf), alternariol mono methylether sulfate (AME-sulf), altenuisol sulfate (ATL-sulf), altenuene sulfate (ALT-sulf) or infectopyrone (INF) is marked with the respective symbol.

Each of the 93 *in vitro* experiments was performed in quintuplicate. Each approach was extracted and diluted 1:100 (*A. infectoria* group approaches) or 1:500 and additionally 1:1000 (*A. alternata, A. tenuissima, A. arborescens* species-group approaches) due to big difference in concentration between the toxins. Each diluted sample solution was injected twice for HPLC-MS/MS analysis. Standard deviations of calculated results of double injections (1:100) and of quadruple injections (1:500 and 1:1000) for each sample were <5%. Quantitative results of the twelve ATs TeA, AOH, AME, ATL, sum(iso)ALT, ATX-I, ATX-II, STTX-III, ALP, TEN, and AA-III produced by each of the 93 isolates are presented in **Figures 3, 4** and are summarized in Supplementary Table S4 as mean values in g/kg or mg/kg of five independent repetitions ± standard deviation (SD). The grouped mean value of each toxin, within each species or hierarchical cluster analysis group, was calculated from the mean values of the respective toxin of all isolates within the respective species- or cluster-group. The grouped mean values were presented in g/kg or mg/kg ± standard error of the mean (SEM) due to different sample sizes (**Table 1**). SPSS was used for the statistical testing of the obtained quantitative data of each AT. Normal distribution of the data was checked using the Shapiro–Wilks-Test and the Kolmogorov–Smirnov-Test at a significance level of 0.01. According to both tests the data was not normally distributed but right-skewed. Logarithmic data transformation did not lead to a normal distribution of the data for all the ATs. Therefore, single-sided variance analysis and the Kruskal–Wallis-Test were applied to check for significant differences between the species-groups, with the result that the distribution between the three species-groups *A. alternata, A. arborescens*, and *A. tenuissima* differed insignificantly. However, between these three and the *A. infectoria* species-group a significant difference (0.05) was observed. Bivariate Pearson Correlation with a two-tailed significance test was applied to produce a sample correlation coefficient (*r*) to evaluate the linear correlation among pairs of *Alternaria* toxins. All reported relationships are statistically significant at a level of 0.01 with positive direction of the relationship meaning that these variables tend to increase together. The strength of the association of the toxin pairs is hereby classified as strong (0.8 < |*r*| < 1) or moderate (0.6 < |*r*| < 0.8). Hierarchical cluster analysis was carried out by SPSS. Ward’s method was applied with Euclidean distance. Mean values of five independent approaches of each isolate of TeA, AOH, AME, ALT, sum(iso)ALT, ATX-I, ATX-II, STTX-III, ALP, TEN, and AA-III and a combination (detected or undetected) of the aforementioned toxins and additionally AOH-sulf, AME-sulf, ALT-sulf, ATL-sulf, and INF were used for calculation. Graphical illustrations were constructed in Microsoft Excel or SPSS.

**Table 1:** Concentrations (mean value ± standard error of the mean) of tenuazonic acid (TeA), alternariol (AOH), alternariol mono methylether (AME), altenuisol (ATL), sum of altenuene and isoaltenuene [Σ(iso)ALT], altertoxin I (ATX-I), altertoxin II (ATX-II), stemphyltoxin III (STTX-III), alterperylenol (ALP), tentoxin (TEN), altenuic acid III (AA-III) in mg/kg produced by the isolates of the *Alternaria alternata*, *A. arborescens*, *A. tenuissima*, and *A. infectoria* species-group and the cluster-groups 1 and 2.

	*Alternaria alternata*	*Alternaria arborescens*	*Alternaria tenuissima*	*Alternaria infectoria*	Cluster1	Cluster2
Number of samples	21	11	35	26	31	62
	Mean value ± standard error of the mean [g/kg^∗^ or mg/kg]						
**TeA**	3.00 ± 0.72^∗^	3.71 ± 1.54^∗^	3.73 ± 0.57^∗^	39.9 ± 39.6	0.269 ± 0.165	3.79 ± 0.46^∗^
**AOH**	3.04 ± 1.49^∗^	3.39 ± 1.36^∗^	2.92 ± 0.91^∗^	84.0 ± 77.1	0.933 ± 0.727	3.31 ± 0.74^∗^
**AME**	1.75 ± 0.47^∗^	1.86 ± 0.53^∗^	1.82 ± 0.38^∗^	68.5 ± 60.4	0.433 ± 0.273	1.98 ± 0.27^∗^
**ATL**	330 ± 105	468 ± 154	267 ± 85	19.2 ± 18.4	10.7^#^	354 ± 64
**Σ(iso)ALT**	91.0 ± 27.2	85.3 ± 37.4	68.9 ± 17.6	10.9 ± 10.3	n.d.	89.4 ± 14.9
**ATX-I**	403 ± 111	327 ± 144	311 ± 49	11.1 ± 4.9	10.7 ± 4.3	370 ± 51
**ATX II**	181 ± 58	166 ± 65	107 ± 22	1.21 ± 0.85	46.4^#^	152 ± 25
**STTX-III**	203 ± 54	184 ± 54	160 ± 32	2.11 ± 1.34	0.157 ± 0.086	192 ± 26
**ALP**	19.5 ± 8.4	8.89 ± 4.31	7.99 ± 2.45	59.6 ± 15.6	58.4 ± 13.8	8.48 ± 1.68
**TEN**	74.3 ± 48.5	58.5 ± 32.9	45.0 ± 10.4	1.31 ± 1.17	n.d.	61.5 ± 18.0
**AA-III**	39.4 ± 20.5	14.9 ± 9.2	20.0 ± 7.3	21.4^#^	n.d.	27.6 ± 8.1

Number of samples	18	9	33	24		
	**Outliers excluded; identified in a Hierarchical cluster analysis** Mean value ± standard error of the mean [g/kg^∗^ or mg/kg]								
**TeA**	3.49 ± 1.11^∗^	4.53 ± 1.77^∗^	3.95 ± 0.58^∗^	0.350 ± 0.212		
**AOH**	3.55 ± 2.42^∗^	4.15 ± 1.56^∗^	3.09 ± 0.96^∗^	0.275 ± 0.178		
**AME**	2.04 ± 0.73^∗^	2.27 ± 0.56^∗^	1.93 ± 0.39^∗^	0.558 ± 0.349		
**ATL**	385 ± 167	573 ± 169	283 ± 89	10.7^#^		
**Σ(iso)ALT**	106 ± 43	104 ± 44	73.1 ± 18.5	n.d.		
**ATX-I**	463 ± 175	395 ± 168	329 ± 50	5.23 ± 1.53		
**ATX II**	211 ± 91	203 ± 74	113 ± 22	n.d.		
**STTX-III**	237 ± 85	225 ± 57	170 ± 33	0.204 ± 0.109		
**ALP**	12.0 ± 4.3	6.26 ± 3.40	7.55 ± 2.49	64.4 ± 16.5		
**TEN**	86.6 ± 79.5	71.5 ± 39.2	47.7 ± 10.8	n.d.		
**AA-III**	46.0 ± 33.5	18.2 ± 11.1	21.2 ± 7.7	n.d.		

*^∗^Value in g/kg; ^#^only one isolate produced the toxin; n.d. (not detected; value < LOD).*

## Results

After 14 days of incubation a visually detectable fungal growth in each test tube was observed: all strains colonized the rice kernels very densely. In general, all targeted 17 non-host specific ATs were produced, with the exception of the host-specific AAL toxins TB1 and TB2. AOH was the most extensively formed toxin up to 28.9 g/kg (GST15a) and 26.9 g/kg (RN02At) followed by TeA with 16.7 g/kg (GST33a), whereas ALP was the least abundant toxin in concentrations up to 315 mg/kg (GH12i) (Supplementary Table S4). INF and biotransformed sulfated forms of AOH, AME, ATL, and ALT were tentatively identified by HRMS (**Table 2**). The sulfates were determined in isolates of all four species-groups. However, INF was found mainly in the *A. infectoria* samples (Supplementary Table S4). Overall, isolates from the species-groups *A. alternata, A. arborescens*, and *A. tenuissima* produced all the targeted ATs with similar mean amounts, whereas *A. infectoria* isolates showed a unique mycotoxin profile with rather low concentrations. In general, 57% of the isolates were low (total toxin production < 5.00 g/kg), 34% medium (total toxin production between 5.00 g/kg and 20.0 g/kg) and 9% high (total toxin amount > 20.0 g/kg) toxin producer strains, whereas medium and high toxin production was observed only in *A. alternata, A. arborescens*, and *A. tenuissima* samples. Due to the big difference in concentration between the toxins, different units (g/kg and mg/kg) were used to represent all results with three significant digits (Supplementary Table S4). No ATs were detected in the control samples, which were incubated but not inoculated.

**Table 2:** Monoisotopic calculated exact masses (EM) and measured accurate masses (AM) of the deprotonated molecules [M-H]^−^ screened for in this study.

	Molecular formula	EM (m/z)	AM (m/z)	Δm/z	Mass error (ppm)
AOH-sulfate ion	[C_14_H_9_O_8_S]^−^	337.0024	337.0029	0.0005	1.5
AME-sulfate ion	[C_15_H_11_O_8_S]^−^	351.0180	351.0186	0.0006	1.7
ATL-sulfate ion	[C_14_H_9_O_9_S]^−^	352.9973	352.9977	0.0004	1.1
ALT-sulfate ion	[C_15_H_15_O_9_S]^−^	371.0442	371.0449	0.0007	1.9
Infectopyrone ion	[C_14_H_15_O_5_]^−^	263.0925	263.0929	0.0004	1.5
ATX-I ion	[C_20_H_15_O_6_]^−^	351.0874	351.0884	0.0010	2.8
ATX-I-H_2_O ion	[C_20_H_13_O_5_]^−^	333.0769	333.0775	0.0006	1.8
ATX-II ion	[C_20_H_13_O_6_]^−^	349.0718	349.0721	0.0003	0.86
ALP ion	[C_20_H_13_O_6_]^−^	349.0718	349.0721	0.0003	0.86
ATX-II+H_2_O ion	[C_20_H_15_O_7_]^−^	367.0823	367.0831	−0.0008	2.2
ALP+H_2_O ion					
ATX-I+OH ion					
ATX-II-H_2_O ion	[C_20_H_11_O_5_]^−^	331.0612	331.0616	0.0004	1.2
STTX-III ion	[C_20_H_11_O_6_]^−^	347.0561	347.0566	0.0005	1.4
STTX-III+H_2_O ion	[C_20_H_13_O_7_]^−^	365.0667	365.0669	0.0002	0.55
ATX-II+OH ion					
ALP+OH ion					
STTX-III+2H_2_O ion	[C_20_H_15_O_8_]^−^	383.0772	383.0769	−0.0003	−0.78
ATX-II+2OH ion					
ALP+2OH ion					
STTX-III-H_2_O ion	[C_20_H_9_O_5_]^−^	329.0455	329.0461	0.0005	1.8

### Mycotoxin Production in Relation to the Classified Species-Group

Ninety-three strains from the four *Alternaria* species-groups *A. alternata, A. arborescens, A. tenuissima*, and *A. infectoria* were chosen for this study to compare their metabolic capability under equal *in vitro* conditions. The chosen conditions triggered the simultaneous production of up to 15 of the 17 targeted mycotoxins by all species-groups, with the exception of most *A. infectoria* group members which produced a maximum of seven mycotoxins simultaneously (**Figures 3, 4**). The average total toxin production (13 ATs) of all isolates was 6.92 g/kg ± 10.0 g/kg (SD), whereas the median value was only half as much (3.11 g/kg) indicating a right-skewed distribution of the data.

#### *Alternaria alternata* Species-Group

Twenty-one strains from the *A. alternata* species-group were chosen for this study. Simultaneous multi-*Alternaria*-toxin production was observed with up to 15 mycotoxins of 17 targeted mycotoxins in three isolates, which were also the highest toxin producers within the *A. alternata* species-group (GST05a; GST15a; RN06Ba). 13 and 12 mycotoxins were detected in four samples each and 11 mycotoxins in three samples. The remaining seven samples contained between one and ten mycotoxins (**Figure 3** and Supplementary Table S4). 43% were low and 43% were medium toxin producers and 14% of the strains produced high amounts of ATs (**Figure 3**). Total toxin production of the isolates ranged between 5.90 mg/kg (GH16a) and 48.5 g/kg (GST15a) (**Figure 3**). The average value was 9.12 g/kg ± 11.9 g/kg (SD) the median value 6.46 g/kg, indicating a right-skewed distribution of toxin quantities formed by the *A. alternata* isolates.

**FIGURE 5:**
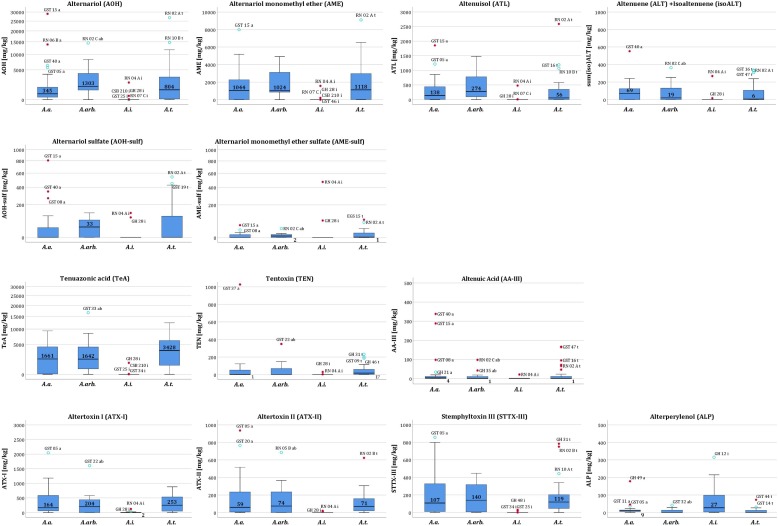
Box-and-whisker plots of *Alternaria* toxin amounts in mg/kg of *A. alternata*, *A. arborescens*, *A. infectoria*, and *A. tenuissima*. Please note different scales due to high differences in concentration between the toxins.

ATX-I was the most frequently produced toxin by 90% of all *A. alternata* isolates. The other perylene quinone derivatives followed with 81% (STTX-III) and 76% (ATX-II and ALP). ATX-I, ATX-II, and STTX-III were produced in amounts up to 2.04 g/kg, 936 mg/kg and 856 mg/kg (GST05a), whereas the maximum determined amount of ALP was only 179 mg/kg (GH49a) (Supplementary Table S4). Mean values were rather low for ATX-I with 403 mg/kg, ATX-II with 181 mg/kg, STTX-III with 203 mg/kg and ALP with 19.5 mg/kg (**Table 1**). However, a right-skewed distribution of the amounts resulted in median values of 164 mg/kg (ATX-I), 59.5 mg/kg (ATX-II), and 107 mg/kg (STTX-III), whereas ALP data distribution was quite symmetrical (median value of 9.40 mg/kg) (**Figure 5**).

**FIGURE 6:**
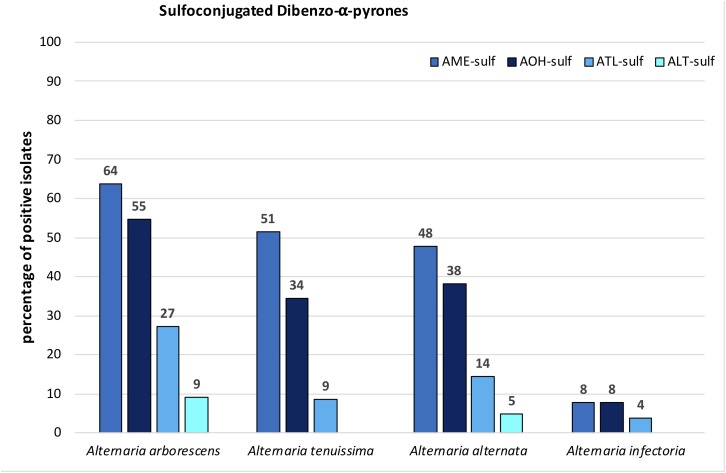
Percentage of *A. arborescens, A. tenuissima, A. alternata*, and *A. infectoria* species-group isolates that biotransformed alternariol mono methylether sulfate (AME-sulf); alternariol sulfate (AOH-sulf), altenuisol sulfate (ATL-sulf), and altenuene sulfate (ALT-sulf).

AOH and AME were produced by 81% of all *A. alternata* isolates and ATL and the sum of ALT and isoALT by 76% and 71%, respectively. Sulfated forms of AOH and AME were biotransformed by 38% and 48% of all *A. alternata* strains, whereas ATL-sulf and ALT-sulf were detected less frequently in three and one samples, respectively (**Figure 6** and Supplementary Table S4). TeA was formed by 76% of all *A. alternata* isolates. AOH concentrations spread extensively from 6.66 mg/kg (RN05Aa) to 28.9 g/kg (GST15a), whereas TeA productions maximum reached only 9.32 g/kg (GST38a) and AME 7.98 g/kg (GST15a) (Supplementary Table S4). However, the AOH mean value (3.04 g/kg) was rather low and comparable with the TeA mean value (3.00 g/kg) (**Table 1**), whereas the AOH median value was ten times lower (345 mg/kg) than the AOH mean, due to extreme outlier isolates. TeA amounts were also right-skewed with a median value half the size (1.67 mg/kg) of the mean (**Figure 5**).

TEN and AA-III were only produced by 57% of the isolates with maximum values of 1.03 g/kg (GST37a) and 338 mg/kg (GST40a) (Supplementary Table S4). Extreme outliers led to a right skewed distribution with low median values (1.59 mg/kg TEN, 3.63 mg/kg AA-III; **Figure 5**) as compared to much higher mean values (74.3 mg/kg TEN, 39.4 mg/kg AA-III; **Table 1**). INF was tentatively detected in one sample (GH49a).

#### *Alternaria arborescens* Species-Group

Eleven strains from the *A. arborescens* species-group were chosen for this study. Simultaneous multi-*Alternaria*-toxin production was observed with up to 15 mycotoxins of 17 targeted mycotoxins in one sample (RN02Ca), which was the second highest toxin producer within the *A. arborescens* species-group. 14 and 13 mycotoxins were detected in one sample each and 12 and 11 mycotoxins in two samples each. The remaining seven samples contained between one and nine mycotoxins (**Figure 3** and Supplementary Table S4). 36% of the strains produced low amounts of ATs, 45% medium amounts and 18% were identified as high toxin producer strains. One isolate did not produce any of the targeted toxins (GST07ab). Therefore, the total toxin production of the isolates ranged between the LOD (0.5–3.0 μg/kg; Supplementary Table S3) and 29.0 g/kg (GST33ab) (**Figure 3**). The average value was 10.3 g/kg ± 10.2 g/kg (SD), the median value 6.84 g/kg, indicating a right skewed distribution of toxin quantities formed by the *A. arborescens* isolates.

AOH was the most frequently produced toxin by 91% of the *A. arborescens* isolates. AME and ATL followed with 82%, whereas ALT and isoALT were only formed by 64% of the *A. arborescens* isolates. Sulfated forms of AOH and AME were detected in 55% and 64% of the isolates, respectively, while ATL-sulf and ALT-sulf were determined less frequently in three and one samples, respectively (**Figure 6** and Supplementary Table S4). TeA was formed by 73% of all *A. arborescens* isolates. TeA and AOH concentrations rose up to 16.7 g/kg (GST33ab) and 14.5 g/kg (GST07ab) (Supplementary Table S4). However, AOH and TeA mean concentrations were rather low (3.39 g/kg, 3.71 g/kg; **Table 1**) but more than twice as high as the respective median value (1.30 mg/kg, 1.64 mg/kg; **Figure 5**).

STTX-III was the most frequently detected perylene quinone derivative in 73% of all samples followed by ATX-I and ATX-II with 64% each and ALP with only 45%. ATX-I, ATX-II, and STTX-III were produced in amounts of up to 1.61 mg/kg (GST22ab), 685 mg/kg (RN05Bab) and 447 mg/kg (GST33ab), respectively, whereas the maximum determined amount of ALP was 41.5 mg/kg (Supplementary Table S4). Mean values were rather low for ATX-I (327 mg/kg), ATX-II (166 mg/kg), STTX-III (184 mg/kg), and ALP (8.89 mg/kg) (**Table 1**). However, a slightly right-skewed distribution of the amounts resulted in comparably lower median values of 204 mg/kg (ATX-I), 74.2 mg/kg (ATX-II), 140 mg/kg (STTX-III), and <1.2 μg/kg (LOD of ALP) (**Figure 5**).

TEN and AA-III were only produced by 45% of the strains with maximum values of 349 mg/kg (GST22ab) and 98.1 mg/kg (RN02Cab). Extreme outliers led to a right-skewed distribution of amounts with low median values below the LOD for TEN (0.9 μg/kg) and AA-III (0.9 μg/kg) (**Figure 5**) as compared to much higher mean values (58.5 mg/kg TEN, 14.9 mg/kg AA-III; **Table 1**). INF was tentatively detected in one sample (GST32ab) (Supplementary Table S4).

#### *Alternaria tenuissima* Species-Group

Thirty-five strains from the *A. tenuissima* species-group were chosen for the analyses. Simultaneous multi-*Alternaria*-toxin production was observed with up to 15 mycotoxins of 17 targeted mycotoxins in two isolates (RN02At and GST16t), which were also the highest and third highest toxin producers within the *A. tenuissima* species-group. Three samples contained 14 and 11 mycotoxins each, five samples 13, 9, and 8 toxins each, seven samples 12 and two samples 10 mycotoxins. In the remaining three samples one, four and five mycotoxins were detected (**Figure 3** and Supplementary Table S4). 40% of the strains were classified as low toxin producer strains, 51% as medium toxin producers and only 9% formed ATs in high amounts. One isolate did not produce any of the targeted toxins (RN06Dt). Therefore, the total toxin production of the isolates ranged between the LOD (0.5–3.0 μg/kg; Supplementary Table S3) and 52.1 g/kg (RN02At) (**Figure 3**). The average value was 9.45 g/kg ± 10.6 g/kg (SD), the median value 8.11 g/kg, indicating an almost symmetrical distribution of toxin quantities formed by the *A. tenuissima* isolates.

ATX-I was the most frequently detected AT formed by 94% of all isolates belonging to the *A. tenuissima* group, closely followed by ATX-II, STTX-III, TeA, AOH, and AME with 91%. TEN was produced by 85% of the samples. ATL and sum(iso)ALT were detected in 77 and 60% of the *A. tenuissima* isolates and sulfated forms of AOH, AME and ATL in 34, 51, and 9%, respectively, (**Figure 6** and Supplementary Table S4). ALP and AA-III were formed less frequently by only 43% and 40% of the isolates and INF was tentatively detected in one sample (GST14t) (Supplementary Table S4).

AOH concentrations spread extensively from 0.405 mg/kg (RN08Dt) to 26.9 g/kg (RN02At), whereas TeA production reached a maximum of 12.4 mg/kg (RN02At) (Supplementary Table S4). Nevertheless, the AOH mean concentration was rather low (2.92 g/kg) but more than three times higher than the median value (804 mg/kg). TeA mean (3.73 g/kg) and median (3.43 g/kg) values were comparable.

ATX-I, ATX-II, and STTX-III concentrations rose up to 879 mg/kg (GST02t), 623 mg/kg (RN02Bt), and 783 mg/kg (GH31t), whereas ALP was only produced up to 72.5 mg/kg (GST44t) (Supplementary Table S4). Mean values were rather low for ATX-I (311 mg/kg), ATX-II (107 mg/kg), STTX-III (160 mg/kg), and ALP (8.89 mg/kg) (**Table 1**). However, a slightly right-skewed distribution of the amounts resulted in comparably lower median values of 253 mg/kg (ATX-I), 69.0 mg/kg (ATX-II), 119 mg/kg (STTX-III), and <1.2 μg/kg (LOD of ALP) (**Figure 5**).

TEN and AA-III were produced with maximum values of 232 mg/kg (GH31t) and 176 mg/kg (GST47t). These outliers led to a right skewed distribution with low median values for TEN (16.7 mg/kg) and AA-III (<LOD of 0.9 μg/kg) **Figure 5**) as compared to higher mean values (45.0 mg/kg TEN, 20.0 mg/kg AA-III; **Table 1**).

#### *Alternaria infectoria* Species-Group

Twenty-six strains from the *A. infectoria* species-group were chosen for this study. All strains displayed only a low toxin production capability. Simultaneous multi-*Alternaria*-toxin production was observed in two outlier isolates with up to 13 and 11 mycotoxins (RN07Ci and RN04Ci). Of the remaining strains one strain produced up to seven mycotoxins (GST01i) and all other samples contained none to five mycotoxins simultaneously (**Figure 4** and Supplementary Table S4). Three isolates did not produce any of the targeted toxins (GH10i; GH19i; GH33i). Therefore, the total toxin production of the isolates ranged between the LOD (0.5–3.0 μg/kg; Supplementary Table S3) and 4.52 g/kg (RN04Ai) (**Figure 4**). The average value was 299 mg/kg ± 910 mg/kg (SD) the median 47 mg/kg.

Two strains (RN04Ai and GH28i) showed a very similar mycotoxin profile to the *A. alternata, A. arborescens*, and *A. tenuissima* species-groups but with rather low amounts of AOH and AME (2.01 g/kg and 1.57 g/kg; RN04Ai) and TeA (1.03 g/kg; GH28i) (Supplementary Table S4). All other targeted ATs and AOH-, AME-, ATL-sulf were formed by the two outliers (**Figure 4**). After exclusion of the isolates RN04Ai and GH28i the total toxin production of the 24 remaining isolates ranged between the LOD (0.5–3.0 μg/kg; Supplementary Table S3) and 328 mg/kg, the average value being 71.4 mg/kg ± 82.9 mg/kg (SD). The mycotoxin profiles were dominated by INF (95%) and ALP (95%) and ATX-I (71%). ALP and ATX-I amounts reached maximum values of 315 mg/kg (GH12i) and 24.8 mg/kg (GST25i) (Supplementary Table S4). STTX-III was produced by 24% of the *A. infectoria* isolates as well as AOH and AME, whereas TeA was detected in only 12% of the isolates. ATL was detected in one sample, while sum(iso)ALT, ATX-II, TEN, and AA-III were produced by none of the isolates. Concentrations of AOH, AME, TeA, and ATL reached levels between 0.206 and 10.7 mg/kg (Supplementary Table S4).

The mean ATX-I (5.23 mg/kg) production of the *A. infectoria* isolates was rather low compared to the *A. alternata, A. arborescens*, and *A. tenuissima* species-groups, whereas the mean ALP concentration (64.4 mg/kg) of the *A. infectoria* isolates was three to six times higher (**Table 1**).

### Mycotoxin Production in Relation to the Chemical Classes and Study Sites

Five of the 93 strains did not produce any of the targeted toxins. The perylene quinone derivatives were most frequently detected in 91% of all samples, followed by the dibenzo-α-pyrone ATs in 71% of all samples, TeA (63%) and ATs that rank among the miscellaneous structures (TEN 49% and AA-III 33%). In contrast, examining the quantified total toxin production, the dibenzo-α-pyrones dominated with a total share of 55%, followed by TeA (37%), whereas the perylene quinone derivatives amount had a rather low share of 7%. Bivariate Pearson Correlation was applied to evaluate the linear correlation among pairs of *Alternaria* toxins (see section “Statistical Analysis”). ATs were likewise detected in 94% of all samples from Germany and in 96% of all samples from the Russian study site.

#### Tenuazonic Acid

Although TeA was detected in only 65% of all samples, it was the most extensively produced AT dominating the total toxin production with a share of 37%. Quantified amounts ranged between the LOD (1.4 μg/kg) and 16.7 g/kg (GST33ab). TeA concentration showed a significantly positive relationship with a moderate association strength with AME. 79% of the strains isolated from Russia produced TeA, while it was only detected in 57% of the samples from German study sites.

#### Dibenzo-α-Pyrone Derivatives

AOH, AME, ATL, and sum(iso)ALT were produced by 71, 70, 59, and 48%, respectively, of all isolates. AOH quantities had almost the same share (35%) as TeA of the total toxin production, AME followed with a share of 19%, ATL with 6% and sum(iso(ALT)) with 2%. Concentrations ranged between the LOD (0.5-3.0 μg/kg; Supplementary Table S3) and 553 mg/kg [sum(iso)ALT; GST40a], 2.59 μg/kg (ATL; RN02At), 9.10 g/kg (AME; RN02At) and 28.9 g/kg (AOH; GST15a). A statistically significant positive relationship between all dibenzo-α-pyrone derivatives was calculated and, in particular, pairs of AOH/AME, AOH/ATL, and AME/ATL showed a strong association. For sum(iso)ALT paired with AOH, AME and ATL each, a moderate association strength was calculated, as well as between AOH and AOH-sulf. With ATs of other chemical classes, a moderate association strength between AOH/AA-III, sum(iso)ALT/AA-III and AME/TeA was observed. AOH, AME, ATL, and sum(iso)ALT were detected in 62, 60, 51, and 43% of all isolates from Germany and, more frequently, in 92, 92, 83 and 58% of all isolates from Russia.

#### Perylene Quinone Derivatives

Even though ATX-I was the most frequently detected AT in 82% of the 93 isolates its share with 4% of the total toxin production was extremely low. ATX-II, STTX-III, and ALP were produced by 61, 69, and 61%, respectively, of all isolates. Concentrations ranged between the respective LODs (0.9-1.2 μg/kg; Supplementary Table S3) and 315 mg/kg (ALP; GH12i), 856 mg/kg (STTX-III; GST05a), 936 mg/kg (ATX-II; GST05a) and 2.04 g/kg (ATX-I; GST05a). A statistically significant positive relationship between the perylene quinone derivatives ATX-I, ATX-II, and STTX-III was found. For ATX-II/STTX-III a strong association was observed, while pairs of ATX-I/ATX-II and ATX-I/STTX-III showed a moderate association. However, with ALP a positive linear relationship was calculated but with no notable association. ATX-I, ATX-II, and STTX-III were detected in 80, 51, and 60% of the German samples and more frequently in 92% of the Russian samples. However, ALP was determined in more samples from Germany (71%) compared with samples from Russia (42%).

#### Miscellaneous Structures

TEN was produced by 52% of all isolates in amounts between its LOD of 0.9 μg/kg and 1.03 g/kg (GST37a) and detected in only 43% of all samples from Germany, but in 79% of all samples from Russia. No statistically significant relationship was observed for TEN with any of the ATs. TEN and AA-III had in sum only a share of 1% of the total toxin production. AA-III was detected in only 34% of the 93 samples in amounts between its LOD of 0.9 μg/kg and 338 mg/kg (GST40a). A positive relationship between pairs of AA-III/AOH and AA-III/sum(iso)ALT with a moderate association strength was measured. AA-III was produced by 38% of the isolates from Germany and 31% of the isolates from Russia. INF occurrence was verified by HRMS with a measured accurate mass of m/z 263.0929 in the negative mode compared to a calculated exact mass of m/z 263.0925 for [M-H]^-^ (mass error 1.5 ppm, **Table 2**). An earlier retention time compared to AA-III and TeA was observed (Supplementary Table S2). Collisional induced mass spectra (MS^2^) in negative mode resulted in two analyte specific ions of m/z 204 and m/z 143. INF was tentatively detected in 25% of all samples. It was found in 35% of all samples from Germany, while only one isolate from Russia produced INF.

#### Modified Toxins

**FIGURE 7:**
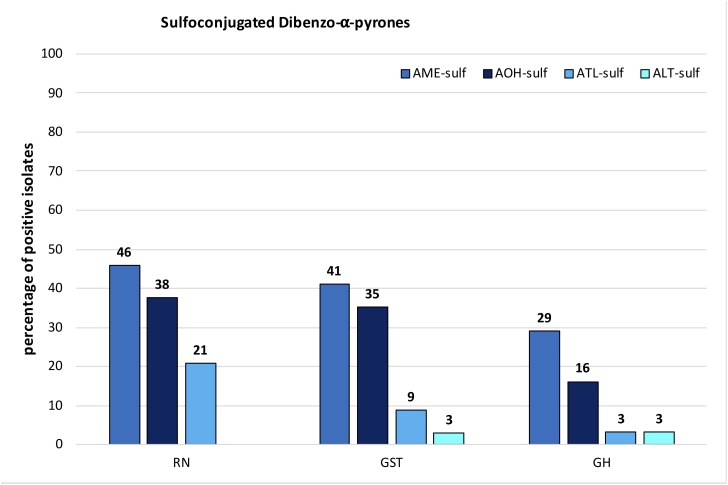
Percentage of species-group isolates originating from Novosibirsk in Russia (RN), Steinfurth in Germany (GST), and Helpt in Germany (GH) that biotransformed alternariol mono methylether sulfate (AME-sulf); alternariol sulfate (AOH-sulf), altenuisol sulfate (ATL-sulf), and altenuene sulfate (ALT-sulf).

Sulfoconjugated forms of AME, AOH, ATL, and ALT were detected in each species-group. Sulfates of other ATs or conjugation with glucose could not be detected with the applied tandem mass spectrometry or high-resolution methods. The ion ratios of the monitored selected ion transitions of the detected sulfates did not display any significant variance (<5%) in any of the cases as well as the monitored retention times (±0.2 min) within all positive samples. All sulfated ATs are more polar than the respective ATs and therefore display an earlier retention time in our method (Supplementary Table S2). Two closely separated peaks at 2.14 and 2.54 min (AOH 3.98 min) were detected in the extracted ion chromatogram of both specific mass transitions for AOH-sulf in negative ESI-LC-MS/MS (m/z 337-m/z 257 and m/z 337-m/z 213). The compounds were thus assumed to represent constitutional isomers of AOH-sulfates, which was tentatively confirmed by HRMS. The same monoisotopic signal at m/z 337.0029 was measured for both compounds. This was observed for samples of all three species-groups (*A. alternata, A. arborescens*, and *A. tenuissima*) containing AOH-sulf in higher amounts. All detected sulfates were confirmed by HRMS with an accuracy of ±5ppm (**Table 2**). In 43% of all samples at least one sulfoconjugated dibenzo-α-pyrone was detected. 15% contained only one, 15% two sulfoconjugated toxins and 8% three. Only in 2 samples (GST15a and GH35ab) all four sulfoconjugated toxins were detected simultaneously. Overall, the most frequently detected biotransformed toxin was AME-sulf with 40% of all samples followed by AOH-sulf with 30%, ATL-sulf with 11% and ALT-sulf with only 2%. Taking a closer look at the four species-groups, 64, 55, and 27% of the *A. arborescens* isolates formed AME-, AOH-, and ATL-sulf, respectively. Also in 51, 34, and 9% of the *A. tenuissima* isolates AME-, AOH-, and ATL-sulf were detected and in 48, 38, and 14% of all *A. alternata* isolates conjugated forms of AME, AOH, and ATL were determined. ALT-sulf was only detected in one *A. arborescens* and one *A. alternata* sample. In contrast to the other species-groups *A. infectoria* isolates hardly produced any of the sulfoconjugated dibenzo-α-pyrones with the exception of the deviating isolates RN04Ai and GH28i, which formed AME-sulf and AOH-sulf and RN04Ai additionally ATL-sulf. (**Figure 6** and Supplementary Table S4). On closer examination of the three study sites – without reference to the species-group – AME-sulf (46%), AOH-sulf (38%), and ATL-sulf (21%) were biotransformed most frequently by 24 *Alternaria* samples isolated in the region of Novosibirsk in Russia (RN). In contrast to this, only 41, 35, and 9% of the 34 *Alternaria* samples isolated in the region of Steinfurth in Germany (GST) formed AME-, AOH-, and ATL-sulf, respectively, and only 29, 16, and 3% of the 31 *Alternaria* strains isolated from the region of Helpt in Germany (GH) (**Figure 7**). ALT-sulf was produced by one sample each of the strains isolated from the two study sites in Germany.

#### Unknown Metabolites

Representative samples of each species-group (GST15a, RN02ab, RN02At, GH34i) were further investigated for unknown metabolites. Especially the extracted ion chromatograms (XICs) of two specific ions for ATX-II showed up to five chromatographically resolved peaks separated from the ATX-II peak (Supplementary Figure S1). ATX-II and ALP share the same mass and a similar fragmentation pattern; therefore, ALP was identified as one of the five peaks in the ATX-II XICs and vice versa. HRMS was carried out to investigate all peaks confirming that four of them shared the same measured accurate mass of 349.0721 (±5ppm) and therefore the same elemental composition (C_20_H_14_O_6_). Likewise, two compounds with the same elemental composition as ATX-I and one as STTX-III were found. Hydrated and dehydrated forms of ATX-I, ATX-II, and STTX-III were detected, and the accurate masses compared to the exact calculated masses (**Table 2**). Possible compounds detected in our study from the literature with a perylene quinone structure are displayed in Supplementary Figure S2.

### Hierarchical Cluster Analysis

**FIGURE 8:**
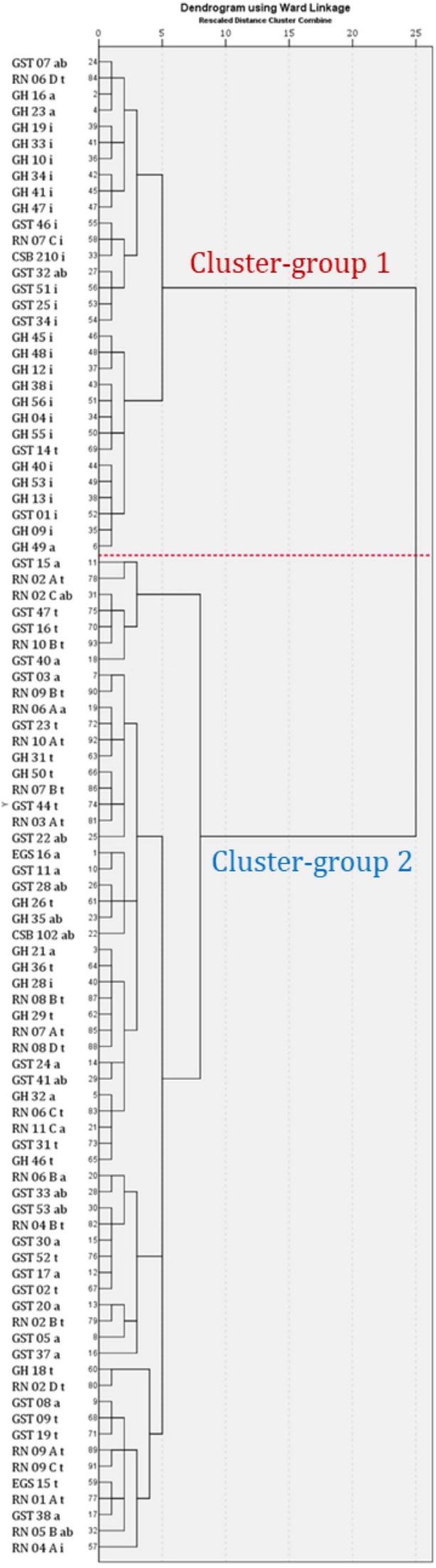
Cluster analysis of 93 metabolite profiles of *Alternaria* species-groups isolates. The dendrogram is based on a hierarchical cluster analysis with Euclidean distance and Ward linkage. Isolates’ names consist of a code of country (first capital letter; G, Germany; R, Russia), region (capital letter; H, Helpt; N, Novosibirsk; St, Steinfurth), identification number and sporulation group (a: *A. alternata*; ab, *A. arborescens*; i, *A. infectoria;* and t, *A. tenuissima*) (Supplementary Table S1).

A hierarchical cluster analysis was used to determine the intrinsic grouping in a set of raw data based on the mycotoxin amounts and mycotoxin combinations of 93 isolates. The resulting clusters were compared with the beforehand morphologically classified species-groups (Supplementary Table S1). The dendrogram indicated two distinct clusters at a distance of 25-8 and 25-5. Cluster 1 comprised 31 of 93 isolates mainly belonging to the *A. infectoria* species-group and cluster 2 comprised 62 of 93, predominantly belonging to the *A. arborescens, A. alternata* and *A. tenuissima* species-group. The AT concentration between the two clusters differed considerably, whereby isolates of the cluster-group 2 produced remarkably higher amounts of the targeted toxins compared to isolates of cluster-group 1 (**Figure 8**).

#### Cluster 1

Cluster 1 contained 24 of 26 isolates belonging to the *A.* *infectoria* group, which produced mostly INF, ALP, and ATX-I and additionally small amounts of TeA, AOH, AME, and STTX-III in a few cases (CSB210.56, GST25i, GST46i, GST51i, RN07Ci, GST34i; Supplementary Table S4). Seven isolates, not classified as *A. infectoria*, were grouped in cluster 1 as well (**Figure 8**). Among them were three isolates from the *A. alternata* species-group, being the weakest toxin producers. ALP was detected in all three samples and additionally ATX-I and INF in one sample. All three isolates originated from fields in the region of Helpt in Germany (GH16a, GH23a, GH49a; **Figure 3**). Further two isolates from the *A. arborescens* species-group were grouped in cluster 1. These isolates were also the weakest toxin producers within the *A. arborescens* group and produced none of the targeted toxins or they produced ALP, ATX-I, ATX-II, and AOH in small amounts and INF (GST07ab, GST32ab; **Figure 3**). The isolates originated from the region of Steinfurth in Germany. Finally, two out of 35 isolates of the *A. tenuissima* species-group clustered also in cluster 1. One did not form any of the targeted toxins, the other only ATX-I, ATX-II, and ALP in small amounts and INF (RN06Dt, GST14t; **Figure 3**). One strain was isolated from the region of Novosibirsk in Russia the other from Steinfurth in Germany.

The isolates in cluster 1 produced generally fewer toxins in smaller amounts compared to cluster 2. INF, ALP, and ATX-I were most frequently determined in 84, 81, and 58% of all cluster-group 1 isolates. The ALP average concentration of 58.4 mg/kg was the highest of all targeted toxins, while the ATX-I average was significantly lower at 10.7 mg/kg. In contrast to this STTX-III was only present in 16% of the samples with a low mean value of 0.157 mg/kg. ATX-II and ATL were detected in one sample in concentrations of 46.4 and 10.7 mg/kg, respectively. TeA was produced by 10% of the isolates with a mean content of 0.269 mg/kg. The dibenzo-α-pyrone derivatives AOH and AME were determined in 19 and 16% of the cluster group 1 samples with mean values of 0.933 mg/kg and 0.433 mg/kg, respectively. TEN, sum(iso)ALT, AA-III and sulfoconjugated AOH, AME, ALT, or ATL were not detected in cluster-group 1 (**Table 1**).

#### Cluster 2

Within cluster 2 no differentiation between *A. arborescens, A. alternata*, and *A. tenuissima* could be observed according to a species-group specific mycotoxin profile (**Figure 8**). Two strains of the *A. infectoria* species-group were found in cluster-group 2 (RN04Ai and GH28i). Both are low toxin producer strains with similar mycotoxin profiles as representatives of the *tenuissima, arborescens* and *alternata* species-groups.

The dibenzo-α-pyrone derivatives were the most frequently and intensively formed ATs in cluster-group 2 with AOH and AME, both formed by 97% of all isolates, followed by ATL (87%) and sum(iso)ALT (73%). The concentration averages ranged from 89.4 mg/kg of sum(iso)ALT to 3.31 g/kg of AOH (**Table 1**). Sulfate conjugates of AOH, AME, ATL, and ALT were detected in 47, 63, 16 and 3% of the samples. The second most frequently detected toxin group was the perylene quinone derivatives group. 95% of all strains in cluster 2 formed STTX-III, 94% ATX-I and 90% ATX-II, while ALP was only detected in 52%. Mean concentrations ranged from 8.48 mg/kg of ALP to 370 mg/kg of ATX-I (**Table 1**). TeA was detected in 92% with the highest mean value of all targeted toxins of 3.79 g/kg. TEN and AA-II were formed by 77 and 52% of the strains with low mean values of 61.5 mg/kg and 27.6 mg/kg, respectively. Further analysis of cluster 2 led to a third cluster containing high toxin producer strains, but with representatives from all three species-groups (**Figure 8**).

## Discussion

### Differentiation of *Alternaria* Strains

*Alternaria* species identification was traditionally based on morphological characteristics of the reproductive structures and sporulation pattern under controlled culture conditions. However, a distinct identification of morphological species groups can be difficult and faulty classification can occur. Where classical morphological methods failed to discriminate among small-spored *Alternaria* species, molecular genetics achieved better results (Roberts et al., 2000; Stewart et al., 2013). Nevertheless, agreement with morphological results is still not satisfying (da Cruz Cabral et al., 2017). Woudenberg et al. (2015) reclassified *Alternaria* section *Alternaria* based on molecular data, whole-genome sequencing and transcriptome analysis to describe only 11 phylogenetic species and one species complex. Several morphospecies (among others *A. tenuissima*) were synonymized under *A. alternata*, while *A. arborescens* remains as a species complex. Such genome and transcriptome comparisons can lead to an important taxonomic change within this fungal genus. However, in addition to this, the secondary metabolite profiles can be a helpful tool to identify and classify *Alternaria* species by means of polyphasic approaches (Frisvad et al., 2008). In previous studies the unique mycotoxin profile of the *A. infectoria* species-group has been described and differentiation from other small-spored *Alternaria* species such as *A. alternata*, *A. tenuissima*, and *A. arborescens* (Andersen et al., 2009; Kahl et al., 2015). Isolates from the *A. infectoria* species-group are described as producers of infectopyrone, 4Z-infectopyrone and novae-zelandin A and B by Christensen et al. (2005). Also, it was stated that members of the *A. infectoria* species group are not able to produce any of the common secondary metabolites such as AOH, TeA, TEN, or ALT but, apart from novae-zelandin, ATX-I and other unidentified altertoxin derivatives (Andersen et al., 2002, 2009, 2015). Contrarily, TeA, AME, AOH, ALT, ATL, TEN, and AA-III were demonstrated to be formed by the *A. infectoria* species-group (Bottalico and Logrieco, 1998; Kahl et al., 2015; Zwickel et al., 2016a). Our results verified the production of AOH, AME, and TeA in rice by the *A. infectoria* isolates but in remarkably lower concentrations (<1 mg/kg) compared to the *A. alternata, A. tenuissima*, and *A. arborescens* species-groups (>1 g/kg). Additionally, the production of ATL, ATX-I, ATX-II, STTX-III, and ALP by the *A. infectoria* species-group was proven. However, we could not confirm the remarkably high amount of STTX-III formed by one *A. infectoria* strain in our previous study (Zwickel et al., 2016a). We also identified the formation of alterperylenol by members of all four investigated *Alternaria* species-groups but most frequently by the majority of *A. infectoria* strains. ALP was produced strongly in three to six times higher concentrations by the *A. infectoria* strains compared to strains of the other species-groups. Furthermore, infectopyrone was identified in 95% of the *A. infectoria* strains and only in one strain in each of the other groups. The hierarchical cluster analysis showed that strains from the *A. infectoria* species-group, which did not produce any INF, might not belong to this group and strains from the other three species-groups, which did produce INF, may be classified as *A. infectoria* group members. In contrast to the studies of (Christensen et al., 2005; Andersen et al., 2015) neither 4Z-infectopyrone nor novae-zelandin A and B could be detected in any of the isolates. The unique mycotoxin pattern of the *A. infectoria* species-group has already been recognized in previous studies (Andersen et al., 2015; Kahl et al., 2015; Zwickel et al., 2016a) and the presence of infectopyrone in this group confirms this substance as a phenotaxonomic marker (Ostenfeld Larsen et al., 2003). ALP has been described for the *Alternaria* genus before (Escriva et al., 2017) but a distinct assignment to the *A. infectoria* species-group has not been made.

Hierarchical cluster analysis was applied, and formed clusters were checked in accordance with the morphologically determined species-groups to verify dissimilarity between the four species-groups and similarity within the species-groups based on the quantitative mycotoxin production and combination of formed mycotoxins. Examining all 93 strains two distinct clusters were revealed. Cluster 1 mostly comprised *A. infectoria* strains and cluster 2 strains of the *A. arborescens, A. alternata*, and *A. tenuissima* species-group. According to this *A. infectoria* could be successfully differentiated from the other three small-spored *Alternaria* species-groups, based on its low mycotoxin production and its unique mycotoxin profile that was mainly defined by infectopyrone, ALP and ATX-I. Seven strains from the other three species-groups clustered with the *A. infectoria* isolates. Despite vigorous growth, two isolates (GST07ab; RN06Dt) did not produce any of the targeted toxins above the LODs (Supplementary Table S4). Reasons for this may be that the produced toxin concentrations were too low to detect or that the two isolates are no longer able to produce any of the targeted toxins after long-term laboratory cultivation. The other five strains share the exclusive production of ALP or in combination with INF, ATX-I, and ATX-II or, in one instance, with AOH. In contrast to ALP, the other toxins were only produced in very low mg/kg amounts in this cluster group compared to strains from the *A. alternata*, *A. arborescens*, or *A. tenuissima* species-groups. According to the resulting mycotoxin cluster the seven strains are likely to be members of the *A. infectoria* species-group. Verification by multi-locus sequence analysis must be carried out. Likewise, two strains classified as *A. infectoria* group members were grouped within cluster 2 producing almost all targeted ATs except INF. Therefore, both strains should not be classified as *A. infectoria* species-group members. In this study, chemotaxonomic profiling could not segregate the members of cluster group 2 which belong to the *A. alternata, A. tenuissima*, and *A. arborescens* species-group. None of the mycotoxin profiles of these species-groups showed a unique species-group specific pattern. The mycotoxin profiles, considering the combination of 17 ATs as well as quantities of 15 ATs, were too similar to distinguish between them. In all three groups low, medium and high toxin producer strains were identified. Their mycotoxin profiles were quantitatively dominated by AOH, TeA, and AME.

### Modified *Alternaria* Toxins

Modified sulfated mycotoxins of AOH, AME, and ATL were produced by all species groups, whereas the respective *A. infectoria* strains, RN04Ai and GH28i, were identified as outliers and possibly have to be considered as not belonging to the *A. infectoria* group. ALT-sulf was produced only by one *A. arborescens* and one *A. alternata* strain. Remarkably, the sulfoconjugates were only detectable in those strains, which also produced the non-conjugated analogs. The sulfoconjugated dibenzo-α-pyrone-derivatives pattern of the *A. alternata, A. arborescens*, and *A. tenuissima* species-groups was quite similar, with AME-sulf being the most frequently detected modified toxin, followed by AOH-sulf and ATL-sulf. For AOH-sulf two chromatographically separable peaks were identified in all three species-groups (*A. alternata, A. arborescens*, and *A. tenuissima*). Pfeiffer et al. (2009) also observed two products of AOH after *in vitro* glucuronidation and elucidated that the glucuronide acid moiety was either bound to the hydroxy group in position 3 or 9, but not 7. The same has also been shown for β-D-glucopyranosides of AOH formed in suspension cultures of tobacco BY-2 cells (Hildebrand et al., 2015). Conjugation to the AOH-9-O position led to the more polar conjugate in both cases (Pfeiffer et al., 2009; Hildebrand et al., 2015). Analogous to this the more polar compound in our study should be AOH-9-O-sulfate followed closely by AOH-3-O-sulfate. In a previous study we identified the formation of sulfoconjugated ATs as a fungal reaction during the later stages of growth (Zwickel et al., 2016a). AOH is suggested to be an important factor supporting the substrate colonization. The ability of AOH to further open wounds of already wounded tomatoes was demonstrated for *A. alternata* strains (Graf et al., 2012) and the high frequency and concentration of AOH in our study may also confirm this suggestion. Hence, after the infection processes of the host cell is completed, the fungus may produce the more water-soluble sulfoconjugates of the comparably more toxic ATs in a self-detoxification process. This is supported by the fact that AOH exhibited an EC_50_ value of 1.7 μg/ml, against L51788 mouse lymphoma cells, while AOH-5-O-sulfate showed lower cytotoxic activity with an EC_50_ value of 4.5 μg/ml (Aly et al., 2008). The fungal vacuole is the primary storage site and trafficking system for metabolites such as amino acids or polyphosphates and carries out degradation processes (Klionsky et al., 1990). In *Phanerochaete velutina*, as a model of a fast-growing filamentous saprotrophic fungus, it was suggested that a complex extended vacuole forms a longitudinal bidirectional transport system (Darrah et al., 2006). Based on this and since the vacuole environment is predominantly aqueous we hypothesize that the formation of sulfoconjugates may be a transport or storage form in the filamentous fungi vacuole system to excrete the toxins for detoxification reasons or substrate colonization support.

### Biosynthesis of *Alternaria* Toxins

Pearson correlation coefficients were calculated for all combinations of ATs to obtain information about the possible various biosynthesis pathways of *Alternaria* toxins which have been studied to a greater and lesser degree. The dibenzo-α-pyrones are biosynthesized via the polyketide pathway. The enzyme-bound polyketide chain is built up by repeating Claisen-condensation of one activated acetyl-CoA starter unit and six malonyl-CoA extender units resulting in a poly-β-keto-intermediate. Following aldol-type cyclization between C2-C7 and C8-C13 catalyzed by heptaketide synthases and subsequent lactonization forms the first aromatic product of the dibenzo-α-pyrones, AOH. Afterwards, AME is built by methylation with *S*-adenosyl methionine as reagent. Hydroxylation of AME results in the formation of desmethylgraphislactone (4-HO-AME) and further reductive cleavage of the lactone ring ALS. Subsequently, oxidative cyclization of ALS leads to dehydroaltenusin and further reduction to ALT or isoALT. ATL can be derived by demethylation and reduction of dehydroaltenusin (Thomas, 1961; Gatenbeck and Hermodsson, 1965; Stinson, 1985; Schäberle, 2016). We consider it also conceivable that after oxidative cleavage of the catechol ring of ALS to a dicarboxylic acid (possibly AA-I) a subsequent lactonization may lead to AA-III and AA-II. Our results identified AOH as the most frequently and extensively formed dibenzo-α-pyrones followed by AME, ATL, (iso)ALT, AME-sulf, AA-III, AOH-sulf, ATL-sulf, and ALT-sulf. ALS was identified in all samples that contained AOH and AME but could not be quantified due to its instability in sample solution (Zwickel et al., 2016b). Bivariate pearson coefficients showed that AOH, AME, and ATL have a strong association, which means they are obviously formed in parallel and their concentrations rise together strongly. (Iso)ALT was only moderately associated with the others dibenzo-α-pyrones and AA-III, which implies the preferred pathway could be the formation of ATL at this stage of growth. AOH also has a moderate connection with AOH-sulf and AA-III, which may be two further metabolic pathway possibilities in addition to the (iso)ALT pathway route. Little is known about the biosynthesis of the C_20_ metabolites of *Alternaria* and *Stemphylium*, which are all derivatives of 4,9-dihydroxy-3,10-perylene quinone. We identified ATX-I as the most frequently and extensively produced perylene quinone which moderately associated with ATX-II and STTX-III. Both epoxides were formed in comparable amounts and showed a strong association meaning that the concentrations rise together strongly. Unexpectedly ALP was preferably formed by *A. infectoria* isolates in comparable amounts and did indeed show a positive linear relationship, but the association strength was extremely low.

### Unknown Perylene Quinone Derivatives

We also detected compounds which share the elemental composition with ATX-I (C_20_H_16_O_6_). Stemphylperylenol (Podlech et al., 2014) and altertoxin IV (Wu et al., 2014) are possible perylene quinone derivates belonging to the dihydroanthracene type. A further detected compound, sharing the elemental composition with ATX-II and ALP (C_20_H_14_O_6_), could be altertoxin V (Bashyal et al., 2014) and another one with the elemental composition of STTX-III (C_20_H_12_O_6_) is likely to be altertoxin III (Stack and Prival, 1986). In addition, we detected monohydrated forms of STTX-III or monohydroxylated forms of ATX-II (C_20_H_14_O_7_) which could be alterlosin I (Stierle et al., 1989) or stemphyltoxin I (Podlech et al., 2014). We also found monohydrated forms of ATX-II or monohydroxylated forms of ATX-I (C_20_H_16_O_7_) which could be stemphytriol (Podlech et al., 2014), 7-epi-8-hydroxy-altertoxin I (Podlech et al., 2014) or alterlosin II, with a dihydroanthracene structure (Stierle et al., 1989) (Supplementary Figure S2). Furthermore, we detected dihydrated forms of STTX-III or dihydroxylated forms of ATX-II (C_20_H_16_O_8_) which so far have not been described to the best of our knowledge. Dehydrated forms of ATX-I (C_20_H_14_O_5_), ATX-II (C_20_H_12_O_5_), and STTX-III (C_20_H_10_O_5_) were also determined. A possible compound named altertoxin VI has only been described for dehydrated ATX-II so far (Bashyal et al., 2014).

### Toxicological Potential of *Alternaria* ssp.

Mycotoxins occur as natural contaminants throughout the entire food and feed chain, hence it is most likely that consumers are exposed to a mixture of various mycotoxins. Due to the aforementioned toxic effects of single ATs, an exposure to multiple mycotoxins probably creates an even stronger adverse effect on human and animal health. Recently, the potency of ATs to inhibit the human type II DNA-topoisomerase and its bacterial equivalent, gyrase, was reported, whereby the human enzyme inhibition increased gradually from ALP (75 μM) over ATX-I (50 μM), AOH, AME, and ATX-II (25 μM) to STTX-III (10 μM). The bacterial enzyme was inhibited increasingly from STTX-III, ATX-I, and ALP (50 μM) followed by ATX-II (25 μM) to AOH and AME (10 μM) (Jarolim et al., 2017). The occurrence of those mycotoxin mixtures has already been linked to stronger adverse impacts and synergistic effects on human and animal health than indicated by a single mycotoxin. Dose-dependent combinatory effects of AOH and ATX-II were demonstrated: low dose combinations revealed additive cytotoxic effects, whereas for the combination of higher concentrations an antagonism was stated (Vejdovszky et al., 2017b). As consumers are mainly exposed to low *Alternaria* toxin amounts (EFSA, 2011), possible additive toxin effects should be of concern. Recently, synergistic estrogenic effects of AOH in combination with toxins produced by *Fusarium* fungi have also been reported (Vejdovszky et al., 2017a). For a reliable and more robust risk assessment, a more precise and realistic exposure assessment can be achieved by means of multianalyte methods to unravel the potency of *Alternaria* species to form ATs. The results of our present study showed that members of the three species-groups *A. alternata*, *A. tenuissima*, and *A. arborescens* have the potential to form at least 15 known mycotoxins simultaneously and especially genotoxic substances such as AOH and AME in very high amounts under similar conditions. Also, sulfoconjugated forms of the dibenzo-α-pyrones were frequently detected. Those biotransformed mycotoxins may be converted into their native forms after consuming contaminated foodstuff. As modified ATs are usually not detected by means of routine analysis methods, an underestimation of the respective AT amount in food and feed is the resulting consequence. Genotoxic ATX-I was detected most frequently, but also ATX-II. STTX-III and ALP were highly represented. Additionally, several other possibly perylene quinone like compounds could be detected. Because of insufficient occurrence data of unknown and even known perylene quinone derivatives, due to the lack of certified standard substances, important information regarding human exposure to these genotoxic compounds is missing. The *A. infectoria* species-group members formed predominantly infectopyrone, about whose biological activity little is known. So far INF has not been found to be cytotoxic against mouse P388 leukemia cells (ID_50_ > 25 μg/mL) (Ostenfeld Larsen et al., 2003). However, highly mutagenic ALP and ATX-I (Stack and Prival, 1986; Jarolim et al., 2017) were also strongly represented and thus the toxicological potential of this species-group should not be underestimated. Our results emphasize the lack of information on the simultaneous exposure to different *Alternaria* mycotoxins.

In summary, the most *Alternaria* strains investigated in this study produced in the vast majority of cases a mycotoxin mixture consisting of up to 15 different components. Strains of the species-group *A. infectoria* differ from the three other species-groups by having a significantly lower toxin level and the specific production of infectopyrone. Further differentiation between the species-groups *A. alternata*, *A. tenuissima*, and *A. arborescens* was not possible, either by the concentration or by specific components or by the composition of the toxin mixture. The results of this study show the widespread ability of *Alternaria* fungi to metabolize the parent toxins to the sulfated forms. The composition of the mycotoxin mixture does not reflect the geographic origin of the strains.

## Conclusion

The ability of phytopathogenic fungi to assert themselves in the phyllosphere of crop plants influences both the infection of the host plant and the competition against other microorganisms in the same habitat. One of the most effective weapons of pathogenic fungi in dealing with other (micro)organisms as well as in colonizing the host plant is the excretion of mycotoxins. This chemical aggression potential is impressively demonstrated in the present study for small-spored *Alternaria* species. They form up to 15 targeted toxins simultaneously under laboratory conditions and it has to be expected that these toxins can be formed at least partially or in lower concentrations in living host plants. *Alternaria* fungi are able to biotransform those mycotoxins in part and thus possess a chemical weapon for better penetration of plant tissues and for successful competition against other microorganisms. Therefore, the occurrence of *Alternaria* fungi in crops is likely to be accompanied by additive or synergistic toxicological effects after consumption of contaminated feed and foodstuffs. Comparison of 93 strains of four different *Alternaria* species-groups revealed the segregation of the *A. infectoria* species-group due to its specific mycotoxin pattern and the overall low concentration levels of the formed toxins. This makes it possible to estimate the different risks associated with *A. infectoria* infections in the field and allows a more differentiated and reliable risk assessment. However, it must be taken into consideration that the toxicological effects of many of these less described toxins of the *A. infectoria* group are not yet known. Fungi of the *Alternaria* species-groups *alternata, arborescens* and *tenuissima* cannot be separated even by the results of our highly specific multianalyte HPLC-MS/MS method. Nevertheless, their hereby displayed capability to produce multiple known and additionally unknown *Alternaria* mycotoxins simultaneously represents a previously underestimated danger. The risk of human co-exposure to multiple mycotoxins raises the concern about their potentially increased toxicological impact on human health.

## Author Contributions

MM and TZ conceived and designed the experiments. TZ prepared the samples for measurement, performed the chemical analysis, and analyzed the data. SK provided the *in vitro* assay and the extraction of mycotoxins. TZ wrote the paper. MM, SK, and MR performed a scientific supervision and manuscript revising.

## Conflict of Interest Statement

The authors declare that the research was conducted in the absence of any commercial or financial relationships that could be construed as a potential conflict of interest.

## References

[B1] Aly A. H. Edrada-Ebel R. Indriani I. D. Wray V. Muller W. E. Totzke F. (2008). Cytotoxic metabolites from the fungal endophyte *Alternaria* sp. and their subsequent detection in its host plant *Polygonum senegalense*. *J. Nat. Prod.* 71 972–980. 10.1021/np070447m 18494522

[B2] Andersen B. Hollensted M. (2008). Metabolite production by different *Ulocladium* species. *Int. J. Food Microbiol.* 126 172–179. 10.1016/j.ijfoodmicro.2008.05.036 18599140

[B3] Andersen B. Kroger E. Roberts R. G. (2002). Chemical and morphological segregation of *Alternaria arborescens*, *A. infectoria* and *A. tenuissima* species-groups. *Mycol. Res.* 106 170–182. 10.1017/S0953756201005263

[B4] Andersen B. Nielsen K. F. Fernandez Pinto V. Patriarca A. (2015). Characterization of *Alternaria* strains from Argentinean blueberry, tomato, walnut and wheat. *Int. J. Food Microbiol.* 196 1–10. 10.1016/j.ijfoodmicro.2014.11.029 25498470

[B5] Andersen B. Sørensen J. L. Nielsen K. F. van den Ende B. G. de Hoog S. (2009). A polyphasic approach to the taxonomy of the *Alternaria infectoria* species-group. *Fungal Genet. Biol.* 46 642–656. 10.1016/j.fgb.2009.05.005 19501664

[B6] Bashyal B. P. Wellensiek B. P. Ramakrishnan R. Faeth S. H. Ahmad N. Leslie Gunatilaka A. A. (2014). Altertoxins with potent anti-HIV activity from *Alternaria tenuissima* QUE1Se, a fungal endophyte of *Quercus emoryi*. *Biorg. Med. Chem.* 22 6112–6116. 10.1016/j.bmc.2014.08.039 25260957 PMC4252765

[B7] Bottalico A. Logrieco A. (1998). “Toxigenic *Alternaria* species of economic importance,” in *Mycotoxins in Agriculture and Food Safety*, eds Sinha K. K. Bhatnagar D. (New York, NY: Marcel Dekker, Inc.), 65–108.

[B8] Christensen K. B. Van Klink J. W. Weavers R. T. Larsen T. O. Andersen B. Phipps R. K. (2005). Novel chemotaxonomic markers of the *Alternaria infectoria* species-group. *J. Agric. Food Chem.* 53 9431–9435. 10.1021/jf0513213 16302758

[B9] da Cruz Cabral L. Rodriguero M. Stenglein S. Fog Nielsen K. Patriarca A. (2017). Characterization of small-spored *Alternaria* from Argentinean crops through a polyphasic approach. *Int. J. Food Microbiol.* 257 206–215. 10.1016/j.ijfoodmicro.2017.06.026 28672174

[B10] Darrah P. R. Tlalka M. Ashford A. Watkinson S. C. Fricker M. D. (2006). The vacuole system is a significant intracellular pathway for longitudinal solute transport in basidiomycete fungi. *Eukaryot. Cell* 5 1111–1125. 10.1128/EC.00026-06 16835455 PMC1489287

[B11] EFSA (2011). Scientific opinion on the risks for animal and public health related to the presence of *Alternaria* toxins in feed and food. *EFSA J.* 9:2407 10.2903/j.efsa.2011.2407

[B12] EFSA Arcella D. Eskola M. Gómez Ruiz J. A. (2016). Dietary exposure assessment to *Alternaria* toxins in the European population. *EFSA J.* 14:e04654 10.2903/j.efsa.2016.4654 PMC13088870 42006026

[B13] Escriva L. Oueslati S. Font G. Manyes L. (2017). *Alternaria* mycotoxins in food and feed: an overview. *J. Food Qual.* 2017:1569748 10.1155/2017/1569748

[B14] European Commission (2017). *SANTE/12089/2016: Guidance Document on Identification of Mycotoxins in Food and Feed.* Available at: https://ec.europa.eu/food/sites/food/files/safety/docs/cs_contaminants_sampling_guid-doc-ident-mycotoxins.pdf

[B15] Fleck S. C. Burkhardt B. Pfeiffer E. Metzler M. (2012). *Alternaria* toxins: altertoxin II is a much stronger mutagen and DNA strand breaking mycotoxin than alternariol and its methyl ether in cultured mammalian cells. *Toxicol. Lett.* 214 27–32. 10.1016/j.toxlet.2012.08.003 22902351

[B16] Fleck S. C. Pfeiffer E. Metzler M. (2014). Permeation and metabolism of *Alternaria* mycotoxins with perylene quinone structure in cultured Caco-2 cells. *Mycotoxin Res.* 30 17–23. 10.1007/s12550-013-0180-0 24173814

[B17] Fleck S. C. Sauter F. Pfeiffer E. Metzler M. Hartwig A. Koberle B. (2016). DNA damage and repair kinetics of the *Alternaria* mycotoxins alternariol, altertoxin II and stemphyltoxin III in cultured cells. *Mutat. Res. Genet. Toxicol. Environ. Mutagen.* 798–799, 27–34. 10.1016/j.mrgentox.2016.02.001 26994491

[B18] Fraeyman S. Croubels S. Devreese M. Antonissen G. (2017). Emerging *Fusarium* and *Alternaria* mycotoxins: occurrence, toxicity and toxicokinetics. *Toxins* 9:228. 10.3390/toxins9070228 28718805 PMC5535175

[B19] Frisvad J. C. Andersen B. Thrane U. (2008). The use of secondary metabolite profiling in chemotaxonomy of filamentous fungi. *Mycol. Res.* 112 (Pt 2), 231–240. 10.1016/j.mycres.2007.08.018 18319145

[B20] Gatenbeck S. Hermodsson S. (1965). Enzymic synthesis of the aromatic product alternariol. *Acta Chem. Scand.* 19 65–71. 10.3891/acta.chem.scand.19-0065

[B21] Graf E. Schmidt-Heydt M. Geisen R. (2012). HOG MAP kinase regulation of alternariol biosynthesis in *Alternaria alternata* is important for substrate colonization. *Int. J. Food Microbiol.* 157 353–359. 10.1016/j.ijfoodmicro.2012.06.004 22726725

[B22] Hasan H. A. H. (1999). Phytotoxicity of pathogenic fungi and their mycotoxins to cereal seedling viability. *Mycopathologia* 148 149–155. 10.1023/A:1007164617175 11189766

[B23] Hildebrand A. A. Kohn B. N. Pfeiffer E. Wefers D. Metzler M. Bunzel M. (2015). Conjugation of the mycotoxins alternariol and alternariol monomethyl ether in tobacco suspension cells. *J. Agric. Food Chem.* 63 4728–4736. 10.1021/acs.jafc.5b00806 25912034

[B24] Jarolim K. Del Favero G. Ellmer D. Stark T. D. Hofmann T. Sulyok M. (2017). Dual effectiveness of *Alternaria* but not *Fusarium* mycotoxins against human topoisomerase II and bacterial gyrase. *Arch. Toxicol.* 91 2007–2016. 10.1007/s00204-016-1855-z 27682608 PMC5364253

[B25] Kahl S. M. Ulrich A. Kirichenko A. A. Muller M. E. H. (2015). Phenotypic and phylogenetic segregation of *Alternaria infectoria* from small-spored *Alternaria* species isolated from wheat in Germany and Russia. *J. Appl. Microbiol.* 119 1637–1650. 10.1111/jam.12951 26381081

[B26] Klionsky D. J. Herman P. K. Emr S. D. (1990). The fungal vacuole: composition, function, and biogenesis. *Microbiol. Mol. Biol. Rev.* 54 266–292. 10.1128/mr.54.3.266-292.1990 PMC372777 2215422

[B27] Lawrence D. Gannibal P. Dugan F. Pryor B. (2014). Characterization of *Alternaria* isolates from the infectoria species-group and a new taxon from *Arrhenatherum*, *Pseudoalternaria arrhenatheria* sp. nov. *Mycol. Prog.* 13 257–276. 10.1007/s11557-013-0910-x

[B28] Lawrence D. P. Gannibal P. B. Peever T. L. Pryor B. M. (2013). The sections of *Alternaria*: formalizing species-group concepts. *Mycologia* 105 530–546. 10.3852/12-249 23687125

[B29] Lee H. B. Patriarca A. Magan N. (2015). *Alternaria* in food: ecophysiology, mycotoxin production and toxicology. *Mycobiology* 43 93–106. 10.5941/myco.2015.43.2.93 26190916 PMC4505009

[B30] Levsen K. Schiebel H. M. Behnke B. Dotzer R. Dreher W. Elend M. (2005). Structure elucidation of phase II metabolites by tandem mass spectrometry: an overview. *J. Chromatogr. A* 1067 55–72. 10.1016/j.chroma.2004.08.165 15844510

[B31] Logrieco A. Moretti A. Solfrizzo M. (2009). *Alternaria* toxins and plant diseases: an overview of origin, occurrence and risks. *World Mycotoxin J.* 2 129–140. 10.3920/WMJ2009.1145

[B32] Meena M. Gupta S. K. Swapnil P. Zehra A. Dubey M. K. Upadhyay R. S. (2017). *Alternaria* toxins: potential virulence factors and genes related to pathogenesis. *Front. Microbiol.* 8:1451 10.3389/fmicb.2017.01451 PMC5550700 28848500

[B33] Mercado Vergnes D. Renard M.-E. Duveiller E. Maraite H. (2006). Identification of *Alternaria* spp. on wheat by pathogenicity assays and sequencing. *Plant Pathol.* 55 485–493. 10.1111/j.1365-3059.2006.01391.x

[B34] Müller M. E. H. Korn U. (2013). *Alternaria* mycotoxins in wheat – A 10 years survey in the Northeast of Germany. *Food Control* 34 191–197. 10.1016/j.foodcont.2013.04.018

[B35] Nemecek G. Cudaj J. Podlech J. (2012). Revision of the structure and total synthesis of altenuisol. *Eur. J. Org. Chem.* 2012 3863–3870. 10.1002/ejoc.201200506

[B36] Nemecek G. Thomas R. Goesmann H. Feldmann C. Podlech J. (2013). Structure elucidation and total synthesis of altenuic acid III and studies towards the total synthesis of altenuic acid II. *Eur. J. Org. Chem.* 2013 6420–6432. 10.1002/ejoc.201300879

[B37] Ostenfeld Larsen T. Perry N. B. Andersen B. (2003). Infectopyrone, a potential mycotoxin from *Alternaria infectoria*. *Tetrahedron Lett.* 44 4511–4513. 10.1016/S0040-4039(03)01018-9

[B38] Ostry V. (2008). *Alternaria* mycotoxins: an overview of chemical characterization, producers, toxicity, analysis and occurrence in foodstuffs. *World Mycotoxin J.* 1 175–188. 10.3920/Wmj2008.X013

[B39] Patriarca A. (2016). *Alternaria* in food products. *Curr. Opin. Food Sci.* 11 1–9. 10.1016/j.cofs.2016.08.007

[B40] Pero R. W. Posner H. Blois M. Harvan D. Spalding J. W. (1973). Toxicity of metabolites produced by the “*Alternaria*”. *Environ. Health Perspect.* 4 87–94. 10.1289/ehp.730487 4198474 PMC1474843

[B41] Pfeiffer E. Schmit C. Burkhardt B. Altemoller M. Podlech J. Metzler M. (2009). Glucuronidation of the mycotoxins alternariol and alternariol-9-methyl ether in vitro: chemical structures of glucuronides and activities of human UDP-glucuronosyltransferase isoforms. *Mycotoxin Res.* 25 3–10. 10.1007/s12550-008-0001-z 23604930

[B42] Podlech J. Fleck S. C. Metzler M. Burck J. Ulrich A. S. (2014). Determination of the absolute configuration of perylene quinone-derived mycotoxins by measurement and calculation of electronic circular dichroism spectra and specific rotations. *Chemistry* 20 11463–11470. 10.1002/chem.201402567 25056998

[B43] Roberts R. G. Reymond S. T. Andersen B. (2000). RAPD fragment pattern analysis and morphological segregation of small-spored *Alternaria* species and species groups. *Mycol. Res.* 104 151–160. 10.1017/S0953756299001690

[B44] Rotem J. (1994). *The Genus Alternaria: Biology, Epidemiology, and Pathogenicity.* St. Paul, MN: American Phytopathological Society.

[B45] Rychlik M. Humpf H. U. Marko D. Danicke S. Mally A. Berthiller F. (2014). Proposal of a comprehensive definition of modified and other forms of mycotoxins including “masked” mycotoxins. *Mycotoxin Res.* 30 197–205. 10.1007/s12550-014-0203-5 24962446 PMC4202116

[B46] Schäberle T. F. (2016). Biosynthesis of α-pyrones. *Beilstein J. Org. Chem.* 12 571–588. 10.3762/bjoc.12.56 27340449 PMC4901931

[B47] Schuchardt S. Ziemann C. Hansen T. (2014). Combined toxicokinetic and in vivo genotoxicity study on *Alternaria* toxins. *External Sci. Rep.* 11:679E 10.2903/sp.efsa.2014.EN-679

[B48] Schwarz C. Tiessen C. Kreutzer M. Stark T. Hofmann T. Marko D. (2012). Characterization of a genotoxic impact compound in *Alternaria alternata* infested rice as altertoxin II. *Arch. Toxicol.* 86 1911–1925. 10.1007/s00204-012-0958-4 23076116

[B49] Solhaug A. Eriksen G. S. Holme J. A. (2016). Mechanisms of action and toxicity of the mycotoxin alternariol: a review. *Basic Clin. Pharmacol. Toxicol.* 119 533–539. 10.1111/bcpt.12635 27341187

[B50] Stack M. E. Mazzola E. P. (1989). Stemphyltoxin III from *Alternaria alternata*. *J. Nat. Prod.* 52 426–427. 10.1021/np50062a042 2746264

[B51] Stack M. E. Prival M. J. (1986). Mutagenicity of the *Alternaria* metabolites altertoxins I, II, and III. *Appl. Environ. Microbiol.* 52 718–722. 3535674 10.1128/aem.52.4.718-722.1986 PMC239103

[B52] Stewart J. E. Andrew M. Bao X. Chilvers M. I. Carris L. M. Peever T. L. (2013). Development of sequence characterized amplified genomic regions (SCAR) for fungal systematics: proof of principle using *Alternaria*, *Ascochyta* and *Tilletia*. *Mycologia* 105 1077–1086. 10.3852/12-287 23449078

[B53] Stierle A. C. Caddlina J. H. Strobel G. A. (1989). Phytotoxins from *Alternaria alternata*, a pathogen of spotted knapweed. *J. Nat. Prod.* 52 42–47. 10.1021/np50061a003

[B54] Stinson E. E. (1985). Mycotoxins - their biosynthesis in *Alternaria*. *J. Food Prot.* 48 80–91. 10.4315/0362-028X-48.1.80 30934497

[B55] Thomas R. (1961). Studies in the biosynthesis of fungal metabolites. 4. Alternariol monomethyl ether and its relation to other phenolic metabolites of *Alternaria tenuis*. *Biochem. J.* 80 234–240. 10.1042/bj0800234 13776530 PMC1243988

[B56] Thomma B. P. (2003). *Alternaria* spp.: from general saprophyte to specific parasite. *Mol. Plant Pathol.* 4 225–236. 10.1046/j.1364-3703.2003.00173.x 20569383

[B57] Tsuge T. Harimoto Y. Akimitsu K. Ohtani K. Kodama M. Akagi Y. (2013). Host-selective toxins produced by the plant pathogenic fungus *Alternaria alternata*. *FEMS Microbiol. Rev.* 37 44–66. 10.1111/j.1574-6976.2012.00350.x 22846083

[B58] Vejdovszky K. Hahn K. Braun D. Warth B. Marko D. (2017a). Synergistic estrogenic effects of *Fusarium* and *Alternaria* mycotoxins in vitro. *Arch. Toxicol.* 91 1447–1460. 10.1007/s00204-016-1795-7 27401186 PMC5316405

[B59] Vejdovszky K. Sack M. Jarolim K. Aichinger G. Somoza M. M. Marko D. (2017b). In vitro combinatory effects of the *Alternaria* mycotoxins alternariol and altertoxin II and potentially involved miRNAs. *Toxicol. Lett.* 267 45–52. 10.1016/j.toxlet.2016.12.011 28007639

[B60] Walravens J. Mikula H. Rychlik M. Asam S. Devos T. Njumbe Ediage E. (2016). Validated UPLC-MS/MS methods to quantitate free and conjugated *Alternaria* toxins in commercially available tomato products and fruit and vegetable juices in Belgium. *J. Agric. Food Chem.* 64 5101–5109. 10.1021/acs.jafc.6b01029 27180605

[B61] Winter C. K. Gilchrist D. G. Dickman M. B. Jones C. (1996). Chemistry and biological activity of AAL toxins. *Adv. Exp. Med. Biol.* 392 307–316. 10.1007/978-1-4899-1379-1_26 8850626

[B62] Woudenberg J. H. Groenewald J. Z. Binder M. Crous P. W. (2013). *Alternaria* redefined. *Stud. Mycol.* 75 171–212. 10.3114/sim0015 24014900 PMC3713888

[B63] Woudenberg J. H. Seidl M. F. Groenewald J. Z. de Vries M. Stielow J. B. Thomma B. P. (2015). *Alternaria* section *Alternaria*: species, *formae speciales* or pathotypes? *Stud. Mycol.* 82 1–21. 10.1016/j.simyco.2015.07.001 26951037 PMC4774270

[B64] Wu W. B. Yue G. C. Huang Q. L. Sun L. L. Zhang W. (2014). A new compound from an endophytic fungus *Alternaria tenuissima*. *J. Asian Nat. Prod. Res.* 16 777–782. 10.1080/10286020.2014.896343 24660902

[B65] Yamagishi D. Akamatsu H. Otani H. Kodama M. (2006). Pathological evaluation of host-specific AAL-toxins and fumonisin mycotoxins produced by *Alternaria* and *Fusarium* species. *J. Gen. Plant Pathol.* 72 323–327. 10.1007/s10327-006-0291-y

[B66] Zwickel T. Kahl S. M. Klaffke H. Rychlik M. Müller M. E. H. (2016a). Spotlight on the underdogs-an analysis of underrepresented *Alternaria* mycotoxins formed depending on varying substrate, time and temperature conditions. *Toxins* 8:344. 10.3390/toxins8110344 27869760 PMC5127140

[B67] Zwickel T. Klaffke H. Richards K. Rychlik M. (2016b). Development of a high performance liquid chromatography tandem mass spectrometry based analysis for the simultaneous quantification of various *Alternaria* toxins in wine, vegetable juices and fruit juices. *J. Chromatogr. A* 1455 74–85. 10.1016/j.chroma.2016.04.066 27283097

