# Supplementary material for: Chemotaxonomy of Mycotoxigenic Small-Spored Alternaria Fungi – Do Multitoxin Mixtures Act as an Indicator for Species Differentiation?
Source: Front Microbiol. 2018 Jul 3;9:1368. doi: 10.3389/fmicb.2018.01368 (PMC6037717; doi:10.3389/fmicb.2018.01368)
Supplement: Supplementary file 1 [file Table_1.pdf]

Chemotaxonomy of Mycotoxigenic Small-Spored Alternaria Fungi – Do Multitoxin Mixtures Act as an Indicator for Species Differentiation?

Theresa Zwickel[1,2*], Sandra M. Kahl[3,4], Michael Rychlik[2] and Marina E. H. Müller[3*]

[1] Federal Institute for Risk Assessment (BfR), Berlin, Germany, [2] Chair of Analytical Food Chemistry, Technical University of Munich, Munich, Germany, [3] Leibniz Centre for Agricultural Landscape Research (ZALF), Müncheberg, Germany, [4] University of Potsdam, Institute of Biochemistry und Biology, Potsdam, Germany

***Correspondence:** Marina E. H. Müller; mmueller@zalf.de

Table-S1: *Alternaria* strains used in this study isolated from winter wheat kernels, with geographical origin and species-group affiliation. The names consist of a code of country (first capital letter; G: Germany, R: Russia), region (capital letter; H: Helpt, N: Novosibirsk, St: Steinfurth), identification number and sporulation group (a: *A. alternata*, ab: *A. arborescens*, i: *A. infectoria* and t: *A. tenuissima*).

Origin / Species-group	Standard culture	Germany region of Helpt	Germany region of Steinfurth	Russia region of Novosibirsk
Alternaria alternata	EGS# 34-016	GH 16a	GST 03a	RN 06Aa
		GH 21a	GST 05a	RN 06Ba
		GH 23a	GST 08a	RN 11Ca
		GH 32a	GST 11a	
		GH 49a	GST 15a	
			GST 17a	
			GST20a	
			GST 24a	
			GST 30a	
			GST 37a	
			GST 38a	
			GST 40a	
Alternaria tenuissima	EGS# 34-015	GH 18t	GST 02t	RN 01At
		GH 26t	GST 09t	RN 02At
		GH 29t	GST 14t	RN 02Bt
		GH 31t	GST 16t	RN 02Dt
		GH 36t	GST 19t	RN 03At
		GH 46t	GST 23t	RN 04Bt
		GH 50t	GST 31t	RN 06Ct
			GST 44t	RN 06Dt
			GST 47t	RN 07At
			GST 52t	RN 07Bt
				RN 08Bt
				RN 08Dt
				RN 09At
				RN 09Bt
				RN 09Ct
				RN 10At
				RN 10Bt

Species-group / Origin	Standard culture	Germany region of Helpt	Germany region of Steinfurth	Russia region of Novosibirsk
Alternaria arborescens	CSB## 102605	GH 35ab	GST 07ab	RN 02Cab
			GST 22ab	RN 05Bab
			GST 28ab	
			GST 32ab	
			GST 33ab	
			GST 41ab	
			GST 53ab	
Alternaria infectoria	CSB## 210.86	GH 04i	GST 01i	RN 04Ai
		GH 09i	GST 25i	RN 07Ci
		GH 10i	GST 34i	
		GH 12i	GST 46i	
		GH 13i	GST 51i	
		GH 19i		
		GH 28i		
		GH 33i		
		GH 34i		
		GH 38i		
		GH 40i		
		GH 41i		
		GH 45i		
		GH 47i		
		GH 48i		
		GH 53i		
		GH 55i		
		GH 56i		

#EGS: Personal collection of Dr. E.G. Simmons
CSB:Centraalbureau voor Schimmelcultures, Fungal Biodiversity Centre (Utrecht)
