# Supplementary material for: Chemotaxonomy of Mycotoxigenic Small-Spored Alternaria Fungi – Do Multitoxin Mixtures Act as an Indicator for Species Differentiation?
Source: Front Microbiol. 2018 Jul 3;9:1368. doi: 10.3389/fmicb.2018.01368 (PMC6037717; doi:10.3389/fmicb.2018.01368)
Supplement: Supplementary file 2 [file Table_2.pdf]

Chemotaxonomy of Mycotoxigenic Small-Spored Alternaria Fungi – Do Multitoxin Mixtures Act as an Indicator for Species Differentiation?

Theresa Zwickel[1,2*], Sandra M. Kahl[3,4], Michael Rychlik[2] and Marina E. H. Müller[3*]

[1] Federal Institute for Risk Assessment (BfR), Berlin, Germany, [2] Chair of Analytical Food Chemistry, Technical University of Munich, Munich, Germany, [3] Leibniz Centre for Agricultural Landscape Research (ZALF), Müncheberg, Germany, [4] University of Potsdam, Institute of Biochemistry und Biology, Potsdam, Germany

***Correspondence:** Marina E. H. Müller; mmueller@zalf.de

Table-S2: Selected ion transitions with optimized collision energies (CE), collision cell exit potential (CXP), declustering potential (DP), ion ratio[#], retention time (Rt) for each analyte and general mass spectrometer[##] and ion source[###] settings

Analyte name	Q1 mass (Da) Quantifier	Q3 mass (Da) Qualifier	DP (volt)	CE (volt)	CXP (volt)	ion ratio	R_t (min)
AAL TB1+TB2 1	504.3	156.8	-80	-34	-11	0.13	3.98
AAL TB1+TB2 2	504.3	112.9	-80	-52	-9		3.98
ALT 1	291.0	229.1	-50	-20	-13	0.56	4.21
ALT 2	291.0	246.9	-50	-28	-11		4.21
AA-III 1	320.9	233.1	-70	-22	-13	0.16	3.72
AA-III 2	320.9	277.0	-70	-18	-15		3.72
ATL 1	272.9	258.1	-90	-32	-15	0.28	3.84
ATL 2	272.9	174.1	-90	-44	-15		3.84
AOH 1	257.0	214.9	-80	-36	-15	0.48	3.98
AOH 2	257.0	147.1	-80	-46	-9		3.98
AME 1	270.9	256.1	-75	-30	-11	0.18	4.62
AME 2	270.9	228.0	-75	-40	-17		4.62
ATX-I 1	351.0	333.1	-70	-14	-11	0.94	4.33
ATX-I 2	351.0	315.0	-70	-22	-9		4.33
ATX-II 1	349.0	330.9	-85	-18	-17	0.21	4.48
ATX-II 2	349.0	313.0	-85	-30	-17		4.48
isoALT 1	291.0	246.9	-65	-18	-13	0.29	4.21
isoALT 2	291.0	229.1	-65	-40	-13		4.21
STTX-III 1	347.0	300.9	-85	-30	-17	0.80	4.51
STTX-III 2	347.0	328.9	-85	-22	-15		4.51
ALP 1	349.0	303	-85	-20	-17	0.40	4.30
ALP 2	349.0	261	-85	-32	-15		4.30
TEN 1	413.0	141.0	-85	-28	-7	0.80	4.37
TEN 2	413.0	270.9	-85	-22	-11		4.37
TeA 1	196.0	138.9	-50	-26	-9	0.84	3.87
TeA 2	196.0	111.9	-50	-32	-7		3.78

Analyte name	Q1 mass (Da) Quantifier	Q3 mass (Da) Qualifier	DP (volt)	CE (volt)	CXP (volt)	ion ratio	R_t (min)
AME-Sulf 1	351.1	271	-50	-28	-11	0.37	3.18
AME-Sulf 2	351.1	256	-50	-40	-17		3.18
AOH-9-O-Sulf 1	337.1	257	-50	-30	-15	0.26	2.14
AOH-3-O-Sulf 1							2.54
AOH-9-O-Sulf 2	337.1	213	-50	-46	-9		2.14
AOH-3-O-Sulf 2							2.54
ATL-Sulf 1	353.1	273	-50	-30	-15	0.34	2.44
ATL-Sulf 2	353.1	230	-50	-44	-15		2.44
ALT-Sulf 1	371.1	291	-45	-14	-19	0.30	2.78
ALT-Sulf 2	371.1	229	-45	-44	-19		2.78
INF 1	263.1	143	-70	-18	-17	0.20	2.31
INF 2	263.1	189	-70	-30	-17		2.31

[#] response of the peak with the lower area divided by the response of the peak with the higher area
[##] MS parameters: Entrance potential: −10 V, collisionally activated dissociation gas: 6 (arbitrary unit)
[###] ESI parameters: curtain gas: 40 psi; spray gas: 35 psi; dry gas: 50 psi; ion spray voltage, −3500 V (0 – 7 min); +3500 (during equilibration time); temperature 550 °C. Ultra-high purity nitrogen (99.999%) was used as gas.
