# Supplementary material for: Chemotaxonomy of Mycotoxigenic Small-Spored Alternaria Fungi – Do Multitoxin Mixtures Act as an Indicator for Species Differentiation?
Source: Front Microbiol. 2018 Jul 3;9:1368. doi: 10.3389/fmicb.2018.01368 (PMC6037717; doi:10.3389/fmicb.2018.01368)
Supplement: Supplementary file 3 [file Table_3.pdf]

Chemotaxonomy of Mycotoxigenic Small-Spored Alternaria Fungi – Do Multitoxin Mixtures Act as an Indicator for Species Differentiation?

Theresa Zwickel[1,2*], Sandra M. Kahl[3,4], Michael Rychlik[2] and Marina E. H. Müller[3*]

[1] Federal Institute for Risk Assessment (BfR), Berlin, Germany, [2] Chair of Analytical Food Chemistry, Technical University of Munich, Munich, Germany, [3] Leibniz Centre for Agricultural Landscape Research (ZALF), Müncheberg, Germany, [4] University of Potsdam, Institute of Biochemistry und Biology, Potsdam, Germany

***Correspondence:** Marina E. H. Müller; mmueller@zalf.de

Table-S3: Limits of detection **(**LODs) and limits of quantification (LOQs) determined according to DIN EN ISO 32645 (10 calibration points, Mandel fitting test at a significance level of 0.01 showed linear regression for all analytes, $R^2>0.96$ for all analytes, confidence interval and t-test at a significance level of 0.05, maximal permissible uncertainty was set to 33.33%)

Analyte	range [µg/L]	LOD [µg/kg]	LOQ [µg/kg]
**ALT**	0.25 – 4.5	3.0	9.5
**AME**	0.25 – 4.5	0.5	1.8
**AOH**	0.50 – 4.5	0.6	2.1
**TEA**	0.25 – 4.5	1.4	4.6
**TEN**	0.25 – 4.5	0.9	3.1
**AAL TB**	0.25 – 4.5	1.7	5.5
**ATX-I**	0.50 – 4.5	1.1	3.7
**ATX-II**	0.50 – 4.5	1.0	3.4
**STTX-III**	0.25 – 4.5	0.9	3.2
**ALP**	0.25 – 4.5	1.2	4.0
**AA-III**	0.25 – 4.5	0.9	3.1
**ATL**	0.50 – 4.5	1.2	3.7
