# Supplementary material for: Chemotaxonomy of Mycotoxigenic Small-Spored Alternaria Fungi – Do Multitoxin Mixtures Act as an Indicator for Species Differentiation?
Source: Front Microbiol. 2018 Jul 3;9:1368. doi: 10.3389/fmicb.2018.01368 (PMC6037717; doi:10.3389/fmicb.2018.01368)
Supplement: Supplementary file 4 [file Table_4.pdf]

Supplementary Material

Chemotaxonomy of Mycotoxigenic Small-Spored Alternaria Fungi – Do Multitoxin Mixtures Act as an Indicator for Species Differentiation?

Theresa Zwickel[1,2*], Sandra M. Kahl[3,4], Michael Rychlik[2] and Marina E. H. Müller[3*]

[1] Federal Institute for Risk Assessment (BfR), Berlin, Germany, [2] Chair of Analytical Food Chemistry, Technical University of Munich, Munich, Germany, [3] Leibniz Centre for Agricultural Landscape Research (ZALF), Müncheberg, Germany, [4] University of Potsdam, Institute of Biochemistry und Biology, Potsdam, Germany

***Correspondence:** Marina E. H. Müller; mmueller@zalf.de

Table-S4: Mean content (n=5) of produced *Alternaria* toxins tenuazonic acid (TeA), alternariol (AOH), alternariol monomethyl ether (AME), altenuisol (ATL), sum of altenuene and isoaltenuene (sum(iso)ALT, altertoxin I (ATX-I), altertoxin II(ATX-II), stemphyltoxin-III (STTX-III), alterperylenol (ALP), tentoxin (TEN) and altenuic acid III (AA-III) by the *Alternaria alternata, A. arborescens, A. tenuissima* and *A. infectoria* isolates at 25 °C after 14 days in rice in g/kg (marked with *) or mg/kg (± standard deviation SD; n.d. not detected = <LOD) and detected sulfated forms (marked with (s))

The names of the strains consist of a code of country (first capital letter; G: Germany, R: Russia), region (capital letter; H: Helpt, N: Novosibirsk, St: Steinfurth), identification number and sporulation group (a: A. alternata, ab: A. arborescens, i: A. infectoria and t: A. tenuissima) (Table-S1).
n.d.: not detected (value <LOD (Table-S3))
\#   only one value above LOQ detected
* mean content in g/kg (all not marked values in mg/kg)
(s): sulfated form of respective Alternaria toxin detected

Table-S4	TeA	AOH	AME	ATL	sum(iso)ALT	ATX-I	ATX-II	STTX-III	ALP	TEN	AA-III	INF
**mean content in g/kg (marked with *) ± SD in g/kg or mean content in mg/kg ± SD in mg/kg**												
***Alternaria alternata* isolates**												
**EGS 34-016**	6.10 ±2.45	163 ±65	35.1 ±14.6 (s)	63.3 ±2.4	124 ±25	2.70 ±0.35	n.d.	225.2 ±0.4	14.0 ±0.1	n.d.	11.1 ±3.6	n.d.
**GH 16a**	n.d.	n.d.	n.d.	n.d.	n.d.	n.d.	n.d.	n.d.	5.9 ±0.6	n.d.	n.d.	n.d.
**GH 21a**	591 ±98	345 ±137 (s)	92.3 ±22.3 (s)	12.2 ±1.3	131 ±18	6.62 ±0.35	6.53 ±0.15	19.4 ±5.0	n.d.	2.64 ±0.99	34.0 ±3.3	n.d.
**GH 23a**	n.d.	n.d.	n.d.	n.d.	n.d.	n.d.	n.d.	n.d.	9.4 ±0.1	n.d.	n.d.	n.d.
**GH 32a**	4.00 ±0.31*	532 ±145	2.16 ±0.55* (s)	33.8 ±16.6	131 ±33	164 ±23	47.6 ±13.2	39.4 ±8.8	n.d.	51.2 ±9.0	n.d.	n.d.
**GH 49a**	n.d.	n.d.	n.d.	n.d.	n.d.	125 ±28	n.d.	n.d.	179 ±39	n.d.	n.d.	d.
**GST 03a**	360 ±111	n.d.	n.d.	n.d.	n.d.	87.6 ±27	13.7 ±4.7	20.9 ±7.4	5.9 ±1.2	n.d.	n.d.	n.d.
**GST 05a**	9.29 ±2.94*	5.67 ±2.18* (s)	5.18 ±1.49* (s)	1.22 ±0.33* (s)	16.4 ±7.5	2.04 ±0.59*	936 ±425	856 ±329	37.3 ±5.8	117 ±34	3.45 ±0.47	n.d.
**GST 08a**	n.d.	924 ±340 (s)	1.12 ±0.49* (s)	327 ±64	120 ±35	267 ±70	83.1 ±35.4	107 ±42	10.1 ±2.7	46.9 ±12.8	98.3 ±24.3	n.d.
**GST 11a**	396 ±44	90.4 ±0.4	5.55 ±1.13	6.19 ±0.72	n.d.	50.2 ±12.5	n.d.	n.d.	45.3 ±0.8	n.d.	n.d.	n.d.
**GST 15a**	9.11 ±2.02*	28.9 ±11.6* (s)	7.98 ±2.33* (s)	1.86 ±0.69* (s)	241 ±52 (s)	78.1 ±33.0	7.02 ±1.83	9.75 ±3.00	6.99 ±2.21	n.d.	289 ±49	n.d.
**GST 17a**	5.89 ±1.12*	3.85 ±1.50* (s)	770 ±363	177 ±80	41.3 ±9.97	412 ±155	59.5 ±17.6	80.1 ±14.1	7.55 ±3.14	105 ±39	19.6 ±0.4	n.d.
**GST20a**	1.66 ±0.62*	240 ±51	1.56 ±0.28*	138 ±42	40.2 ±9.9	1.18 ±0.50*	765 ±194	798 ±101	12.5 ±2.3	56.5 ±17.8	8.16 ±1.62	n.d.
**GST 24a**	4.12 ±1.04*	184 ±49	1.01 ±0.32*	143 ±41	68.7 ±13.2	440 ±179	225 ±47	336 ±40	23.9 ±4.5	n.d.	n.d.	n.d.
**GST 30a**	3.12 ±0.98*	1.18 ±0.48*	2.26 ±0.76*	440 ±131	156 ±60	588 ±288	124 ±38	143 ±47	21.8 ±6.2	1.50 ±0.45	11.1 ±4.1	n.d.
**GST 37a**	3.32 ±1.08*	161 ±32	1.04 ±0.16*	114 ±27	25.9 ±0.9	916 ±224	323 ±91	263 ±66	9.90 ±1.58	1.03 ±0.30*	n.d.	n.d.
**GST 38a**	9.32 ±1.71*	636 ±114	4.13 ±0.98* (s)	596 ±129	95.8 ±20.0	693 ±132	283 ±89	327 ±139	n.d.	20.6 ±8.1	3.63[#]	n.d.
**GST 40a**	n.d.	6.29 ±1.95* (s)	2.48 ±1.01* (s)	856 ±334	553 ±111	61.3 ±9.7	6.09 ±0.54	10.2 ±5.05	12.8 ±3.2	n.d.	338 ±136	n.d.
**RN 06Aa**	4.35 ±1.06*	6.66 ±1.95	6.17 ±0.72	n.d.	n.d.	100 ±16	170 ±62	350 ±130	n.d.	0.37 ±0.10	n.d.	n.d.
**RN 06Ba**	6.33 ±1.38*	13.9 ±1.7* (s)	4.70 ±1.14* (s)	660 ±109 (s)	75.1 ±17.4	717 ±209	516 ±208	362 ±155	8.62 ±2.53	123 ±34	5.23 ±1.66	n.d.
**RN 11Ca**	1.05 ±0.36*	687 ±168 (s)	2.16 ±0.64* (s)	297 ±75	92.4 ±11.9	529 ±154	234 ±73	313 ±81	n.d.	8.08 ±0.30	5.75[#]	n.d.

Table-S4	TeA	AOH	AME	ATL	sum(iso)ALT	ATX-I	ATX-II	STTX-III	ALP	TEN	AA-III	INF
mean content in g/kg (marked with *) ± SD in g/kg or mean content in mg/kg ± SD in mg/kg												
Alternaria arborescens isolates												
**CSB 102605**	1.52 ±0.48*	2.45 ±0.83* (s)	985 ±353 (s)	1.48 ±0.51* (s)	27.9 ±6.9	n.d.	14.2 ±0.1	365 ±109	n.d.	n.d.	2.61 ±0.62	n.d.
**GH 35ab**	2.67 ±0.38*	985 ±353 (s)	976 ±445 (s)	22.7 ±7.8 (s)	253 ±70 (s)	n.d.	n.d.	n.d.	n.d.	n.d.	42.7 ±11.4	n.d.
**GST 07ab**	n.d.	n.d.	n.d.	n.d.	n.d.	n.d.	n.d.	n.d.	n.d.	n.d.	n.d.	n.d.
**GST 22ab**	2.51 ±1.20*	585 ±286 (s)	745 ±45 (s)	166 ±21	n.d.	1.61 ±0.44*	236 ±95	267 ±110	11.7 ±2.2	349 ±157	n.d.	n.d.
**GST 28ab**	938 ±13	979 ±1	1.02 ±0.05*	136 ±27	n.d.	n.d.	n.d.	12.1 ±2.2	n.d.	n.d.	n.d.	n.d.
**GST 32ab**	n.d.	22.3 ±6.7	n.d.	n.d.	n.d.	38.1 ±17.2	45.1 ±6.3	n.d.	41.5 ±9.2	n.d.	n.d.	d.
**GST 33ab**	16.7 ±4.4*	8.21 ±1.04* (s)	2.36 ±0.57* (s)	321 ±76	14.1 ±3.7	525 ±252	367 ±143	447 ±137	29.2 ±5.3	9.68 ±1.42	1.21 ±0.19	n.d.
**GST 41ab**	1.64 ±0.41*	5.96 ±1.80* (s)	1.14 ±0.52* (s)	274 ±107	95.1 ±20.7	353 ±148	234 ±90	269 ±117	n.d.	n.d.	n.d.	n.d.
**GST 53ab**	8.45 ±2.21*	2.30 ±0.70*	3.88 ±0.94* (s)	867 ±219	165 ±52	288 ±74	74.2 ±12.1	96.9 ±18.5	1.2 ±0.4	112 ±4	19.5 ±6.8	n.d.
**RN 02Cab**	6.31 ±1.45*	14.5 ±2.04* (s)	4.91 ±0.18* (s)	1.17 ±0.14* (s)	364 ±107	583 ±96	213 ±39	140 ±46	14.4 ±1.8	147 ±50	98.1 ±17.7	n.d.
**RN 05Bab**	n.d.	1.30*#	4.45 ±0.63*	693 ±73	18.5 ±0.7	204 ±78	685 ±20	424 ±69	n.d.	26.4 ±1.2	n.d.	n.d.

Table-S4	TeA	AOH	AME	ATL	sum(iso)ALT	ATX-I	ATX-II	STTX-III	ALP	TEN	AA-III	INF
mean content in g/kg (marked with *) ± SD in g/kg or mean content in mg/kg ± SD in mg/kg												
Alternaria tenuissima isolates												
**EGS 34-015**	1.70 ±0.71*	65.2 ±1.4 (s)	1.14 ±0.02* (s)	n.d.	109 ±1	113 ±4	28.8 ±7.5	340 ±113	n.d.	9.92 ±3.00	6.43 ±0.43	n.d.
**GH 18t**	5.56 ±0.60*	1.05 ±0.18*	594 ±26	24.4 ±6.0	n.d.	57.6 ±6.8	38.2 ±11.1	62.8 ±11.9	n.d.	n.d.	n.d.	n.d.
**GH 26t**	669 ±175	153 ±12 (s)	24.0 ±2.5	27.2 ±6.0	18.8 ±5.9	n.d.	n.d.	n.d.	n.d.	105 ±25	n.d.	n.d.
**GH 29t**	1.11 ±0.24*	23.8 ±10.9	115 ±23 (s)	n.d.	5.66 ±0.83	20.4 ±8.2	10.6 ±0.6	74.3 ±8.7	n.d.	29.6 ±7.9	n.d.	n.d.
**GH 31t**	6.20 ±1.23*	469 ±167	2.48 ±0.84* (s)	n.d.	n.d.	124 ±17	244 ±28	783 ±147	n.d.	232 ±60	n.d.	n.d.
**GH 36t**	936 ±296	325 ±95 (s)	152 ±67 (s)	21.4 ±5.7	51.2 ±24.8	150 ±10	70.6 ±5.9	66.2 ±2.3	n.d.	50.8 ±19.5	23.7 ±9.6	n.d.
**GH 46t**	6.11 ±2.78*	1.24 ±0.12*	4.39 ±1.67* (s)	543 ±131	9.38 ±3.70	701 ±149	176 ±27	186 ±20	n.d.	187 ±37	n.d.	n.d.
**GH 50t**	3.72 ±0.78*	1.78 ±0.15*	1.18 ±0.12* (s)	128 ±27	n.d.	860 ±78	141 ±39	244 ±54	24.8 ±4.6	31.0 ±12.0	n.d.	n.d.
**GST 02t**	7.73 ±0.94*	600 ±28 (s)	1.12 ±0.03*	151 ±6	24.7 ±5.8	879 ±9	185 ±7	197 ±11	5.05 ±0.63	11.1 ±1.2	71.8 ±3.0	n.d.
**GST 09t**	6.65 ±0.46*	804 ±19 (s)	1.53 ±0.18* (s)	247 ±57	96.9 ±0.4	415 ±99	67.3 ±15.1	72.2 ±22.1	7.92 ±2.44	200 ±52	14.3 ±2.9	n.d.
**GST 14t**	n.d.	n.d.	n.d.	n.d.	n.d.	42.0 ±1.1	46.4 ±7.6	n.d.	30.3 ±4.7	n.d.	n.d.	d.

Table-S4	TeA	AOH	AME	ATL	sum(iso)ALT	ATX-I	ATX-II	STTX-III	ALP	TEN	AA-III	INF
**mean content in g/kg (marked with *) ± SD in g/kg or mean content in mg/kg ± SD in mg/kg**												
***Alternaria tenuissima* isolates**												
**GST 16t**	4.33 ±0.98*	11.7 ±3.7* (s)	5.06 ±0.97* (s)	1.19 ±0.09* (s)	308 ±68	195 ±67	36.4 ±2.6	38.0 ±11.1	3.72 ±0.90	25.8 ±4.3	95.4 ±26.8	n.d.
**GST 19t**	1.12 ±0.30*	6.71 ±0.04* (s)	1.73 ±0.08* (s)	295[#]	198 ±75	123 ±19	30.3 ±4.4	62.8 ±9.9	3.83 ±2.73	107 ±22	47.0 ±10.8	n.d.
**GST 23t**	3.98 ±1.27*	5.76 ±0.09*	205 ±4	n.d.	n.d.	568 ±117	142 ±32	206 ±63	n.d.	50.6 ±1.1	n.d.	n.d.
**GST 31t**	7.20 ±1.56*	3.09 ±1.12*	3.48 ±0.71* (s)	511 ±84	164 ±23	793 ±173	252 ±56	202 ±70	n.d.	67.2 ±13.9	3.25 ±0.63	n.d.
**GST 44t**	2.46*[#]	975[#]	329[#]	56.0[#]	n.d.	500[#]	n.d.	182[#]	72.5[#]	27.0[#]	n.d.	n.d.
**GST 47t**	6.57 ±1.28*	6.41 ±0.29*	3.81 ±0.85* (s)	583 ±121	329 ±91	348 ±111	117 ±14	150 ±13	9.92 ±0.34	116 ±28	167 ±17	n.d.
**GST 52t**	3.43 ±0.52*	2.61 ±0.37*	3.41 ±0.59*	479 ±96	103 ±18	845 ±171	254 ±35	319 ±79	16.6 ±3.0	2.90 ±0.71	27.8 ±5.2	n.d.
**RN 01At**	3.08 ±0.69*	3.27 ±0.43* (s)	4.37 ±0.80* (s)	74.4 ±12.5	118 ±23	87.9 ±12.7	33.9 ±10.4	62.3 ±16.4	n.d.	1.31 ±0.36	8.29 ±1.25	n.d.
**RN 02At**	12.4 ±2.3*	26.9 ±5.57* (s)	9.10 ±2.03* (s)	2.59 ±0.50* (s)	336 ±69	398 ±122	171 ±43	119 ±21	16.2 ±1.7	38.2 ±6.9	66.2 ±13.2	n.d.
**RN 02Bt**	5.12 ±0.38*	6.78 ±1.22	14.0 ±2.3	130 ±8	n.d.	572 ±253	623 ±202	750 ±103	26.5 ±4.2	69.0 ±22.9	n.d.	n.d.
**RN 02Dt**	3.31 ±1.23*	334 ±88 (s)	2.26 ±0.70* (s)	381 ±138	19.7 ±3.8	611 ±170	99.5 ±12.2	89.7 ±13.7	21.9 ±2.6	n.d.	n.d.	n.d.
**RN 03At**	n.d.	1.73 ±0.78	2.27 ±1.17	n.d.	n.d.	177 ±51	144 ±37	241 ±45	19.6 ±2.8	16.7 ±2.6	n.d.	n.d.
**RN 04Bt**	5.41 ±0.92*	6.61 ±1.23*	6.53 ±1.09* (s)	22.3 ±4.3	78.5 ±23.3	253 ±60	99.3 ±13.2	119 ±17	6.75 ±0.76	109 ±15	5.18 ±0.54	n.d.
**RN 06Ct**	4.42 ±0.57*	1.11 ±0.06*	2.34 ±0.29* (s)	320 ±52.5	205 ±22	304 ±110	49.3 ±20.5	53.9 ±15.8	n.d.	43.1 ±18.4	n.d.	n.d.
**RN 06Dt**	n.d.	n.d.	n.d.	n.d.	n.d.	n.d.	n.d.	n.d.	n.d.	n.d.	n.d.	n.d.
**RN 07At**	718 ±227	595 ±167	148 ±50	11.2 ±1.5	n.d.	406 ±75	144 ±5	181 ±44	n.d.	9.09 ±1.80	n.d.	n.d.
**RN 07Bt**	6.81 ±1.01*	3.64 ±1.03* (s)	2.54 ±0.77* (s)	388 ±119	n.d.	578 ±120	199 ±45	203 ±72	14.1 ±2.8	7.33 ±1.36	n.d.	n.d.
**RN 08Bt**	5.93 ±0.47	1.06 ±0.07	4.34 ±0.40	0.550 ±0.046	0.110 ±0.024	0.459 ±0.151	0.131 ±0.040	0.176 ±0.028	n.d.	0.126 ±0.028	n.d.	n.d.
**RN 08Dt**	3.26 ±0.71	0.405 ±0.098	2.28 ±0.71	0.254 ±0.091	n.d.	0.113 ±0.036	0.0781 ±0.0217	0.0963 ±0.0357	n.d.	n.d.	n.d.	n.d.
**RN 09At**	6.84±1.87	2.21 ±0.55	8.44 ±0.73	1.26 ±0.18	0.158 ±0.060	0.786 ±0.214	0.133 ±0.049	0.182 ±0.047	n.d.	0.0100 ±0.002	n.d.	n.d.
**RN 09Bt**	19.5[#]	n.d.	n.d.	n.d.	n.d.	2.21[#]	1.51[#]	1.38[#]	n.d.	0.298[#]	n.d.	n.d.
**RN 09Ct**	7.55 ±2.33	6.78 ±0.80	5.65 ±1.07	0.751 ±0.182	0.282 ±0.114	0.774 ±0.019	0.0778 ±0.0198	0.0871 ±0.0200	n.d.	0.342 ±0.169	0.0604 ±0.0174	n.d.
**RN 10At**	9.77 ±3.36*	1.04 ±0.32*	889 ±233	95.8 ±28.8	n.d.	469 ±30	306 ±59	443 ±117	n.d.	12.6 ±1.5	n.d.	n.d.
**RN 10Bt**	9.91 ±3.63*	14.8 ±5.8* (s)	4.78 ±1.02* (s)	1.07 ±0.34* (s)	238 ±76	304 ±84	116 ±3	155 ±24	n.d.	14.6 ±5.4	165 ±73	n.d.

Table-S4	TeA	AOH	AME	ATL	sum(iso)ALT	ATX-I	ATX-II	STTX-III	ALP	TEN	AA-III	INF
**mean content in g/kg (marked with *) ± SD in g/kg or mean content in mg/kg ± SD in mg/kg**												
Alternaria infectoria **isolates**												
**CSB 210.86**	4.01 ±0.89	4.20 ±0.19	4.34 ±0.98	n.d.	n.d.	n.d.	n.d.	n.d.	n.d.	n.d.	n.d.	d.
**GH 04i**	n.d.	n.d.	n.d.	n.d.	n.d.	0.403 ±0.157	n.d.	n.d.	48.9 ±4.4	n.d.	n.d.	d.
**GH 09i**	n.d.	n.d.	n.d.	n.d.	n.d.	2.24 ±0.02	n.d.	n.d.	107 ±10	n.d.	n.d.	d.
**GH 10i**	n.d.	n.d.	n.d.	n.d.	n.d.	n.d.	n.d.	n.d.	n.d.	n.d.	n.d.	n.d.
**GH 12i**	n.d.	n.d.	n.d.	n.d.	n.d.	11.2 ±1.6	n.d.	1.30 ±0.39	315 ±68	n.d.	n.d.	d.
**GH 13i**	n.d.	n.d.	n.d.	n.d.	n.d.	1.56 ±0.60	n.d.	n.d.	94.6 ±34.8	n.d.	n.d.	d.
**GH 19i**	n.d.	n.d.	n.d.	n.d.	n.d.	n.d.	n.d.	n.d.	n.d.	n.d.	n.d.	n.d.
**GH 28i**	1.03 ±0.15*	173 ±20 (s)	202 ±64 (s)	8.75 ±0.58	15.4 ±0.2	39.3 ±6	12.8 ±0.4	21.5 ±1.5	n.d.	30.2 ±9.3	n.d.	n.d.
**GH 33i**	n.d.	n.d.	n.d.	n.d.	n.d.	n.d.	n.d.	n.d.	n.d.	n.d.	n.d.	n.d.
**GH 34i**	n.d.	n.d.	n.d.	n.d.	n.d.	n.d.	n.d.	n.d.	9.39 ±8.44	n.d.	n.d.	d.
**GH 38i**	n.d.	n.d.	n.d.	n.d.	n.d.	24.5#	n.d.	n.d.	44.5 ±21.3	n.d.	n.d.	d.
**GH 40i**	n.d.	n.d.	n.d.	n.d.	n.d.	14.8 ±1.9	n.d.	n.d.	151 ±15	n.d.	n.d.	d.
**GH 41i**	n.d.	n.d.	n.d.	n.d.	n.d.	n.d.	n.d.	n.d.	20.4 ±5.9	n.d.	n.d.	d.
**GH 45i**	n.d.	n.d.	n.d.	n.d.	n.d.	3.86 ±0.48	n.d.	0.0533#	122 ±14	n.d.	n.d.	d.
**GH 47i**	n.d.	n.d.	n.d.	n.d.	n.d.	n.d.	n.d.	n.d.	33.6 ±24.1	n.d.	n.d.	d.
**GH 48i**	n.d.	n.d.	n.d.	n.d.	n.d.	8.01 ±1.45	n.d.	2.24#	215 ±37	n.d.	n.d.	d.
**GH 53i**	n.d.	n.d.	n.d.	n.d.	n.d.	8.20 ±3.16	n.d.	n.d.	160 ±46	n.d.	n.d.	d.
**GH 55i**	n.d.	n.d.	n.d.	n.d.	n.d.	4.90#	n.d.	n.d.	16.8 ±4.2	n.d.	n.d.	d.
**GH 56i**	n.d.	n.d.	n.d.	n.d.	n.d.	2.25 ±0.99	n.d.	n.d.	43.1 ±17.8	n.d.	n.d.	d.
**GST 01i**	n.d.	n.d.	n.d.	n.d.	n.d.	4.35 ±1.30	n.d.	n.d.	98.4 ±25.1	n.d.	n.d.	d.
**GST 25i**	3.15 ±0.62	0.885 ±0.198	0.206#	n.d.	n.d.	24.8 ±2.5	n.d.	0.913#	52.4 ±33.0	n.d.	n.d.	d.
**GST 34i**	1.19 ±0.17	n.d.	n.d.	n.d.	n.d.	13.5#	n.d.	0.355 ±0.065	4.64 ±1.86	n.d.	n.d.	d.
**GST 46i**	n.d.	0.291 ±0.036	7.40#	n.d.	n.d.	n.d.	n.d.	n.d.	3.44 ±0.76	n.d.	n.d.	d.
**GST 51i**	n.d.	0.383 ±0.045	0.315#	n.d.	n.d.	0.993#	n.d.	n.d.	4.28#	n.d.	n.d.	d.
**RN 04Ai**	n.d.	2.01 ±0.31* (s)	1.57 ±0.05* (s)	479 ±21 (s)	268 ±71	124 ±58	18.7 ±4.7	28.5 ±8.4	4.00 ±0.21	3.74 ±0.68	21.4 ±3.8	n.d.
**RN 07Ci**	n.d.	0.819 ±0.246	1.17 ±0.20	10.7 ±1.2	n.d.	n.d.	n.d.	n.d.	1.05 ±0.01	n.d.	n.d.	d.
