# Supplementary material for: Chemotaxonomy of Mycotoxigenic Small-Spored Alternaria Fungi – Do Multitoxin Mixtures Act as an Indicator for Species Differentiation?
Source: Front Microbiol. 2018 Jul 3;9:1368. doi: 10.3389/fmicb.2018.01368 (PMC6037717; doi:10.3389/fmicb.2018.01368)
Supplement: Supplementary file 5 [file Image_1.pdf]

Chemotaxonomy of Mycotoxigenic Small-Spored Alternaria Fungi – Do Multitoxin Mixtures Act as an Indicator for Species Differentiation?

Theresa Zwickel[1,2*], Sandra M. Kahl[3,4], Michael Rychlik[2] and Marina E. H. Müller[3*]

[1] Federal Institute for Risk Assessment (BfR), Berlin, Germany, [2] Chair of Analytical Food Chemistry, Technical University of Munich, Munich, Germany, [3] Leibniz Centre for Agricultural Landscape Research (ZALF), Müncheberg, Germany, [4] University of Potsdam, Institute of Biochemistry und Biology, Potsdam, Germany

***Correspondence:** Marina E. H. Müller; mmueller@zalf.de

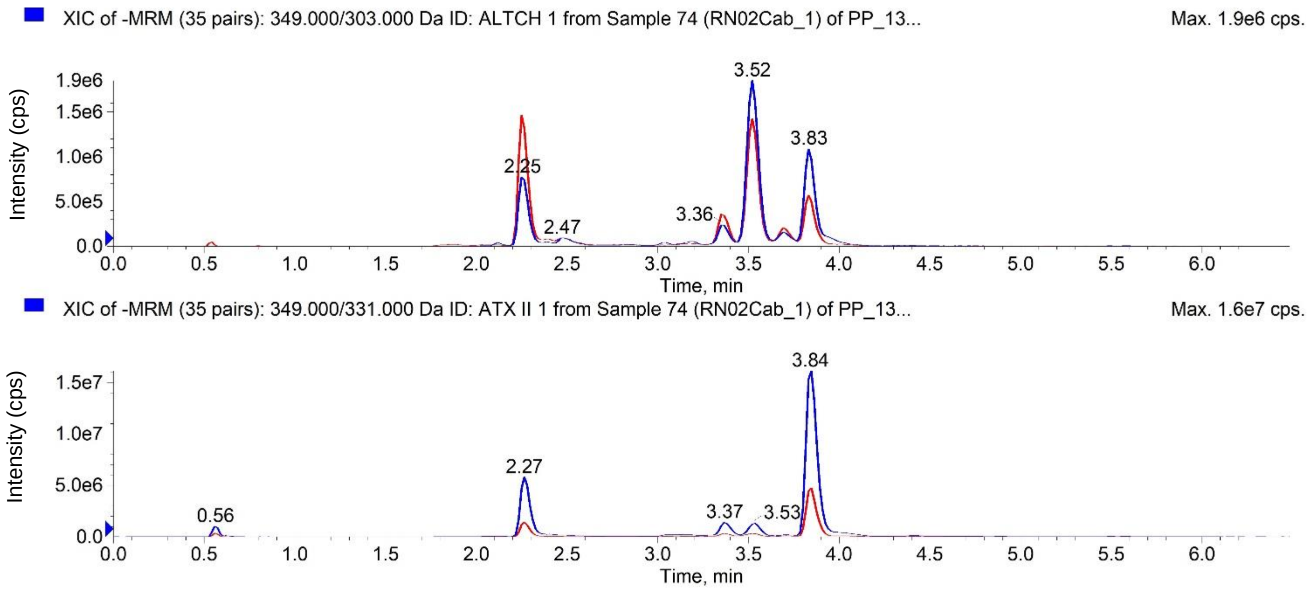

Figure-S1: Extracted ion chromatogram of alterperylenol (above) and altertoxin-II (below)
