# Supplementary material for: Chemotaxonomy of Mycotoxigenic Small-Spored Alternaria Fungi – Do Multitoxin Mixtures Act as an Indicator for Species Differentiation?
Source: Front Microbiol. 2018 Jul 3;9:1368. doi: 10.3389/fmicb.2018.01368 (PMC6037717; doi:10.3389/fmicb.2018.01368)
Supplement: Supplementary file 6 [file Image_2.pdf]

Chemotaxonomy of Mycotoxigenic Small-Spored Alternaria Fungi – Do Multitoxin Mixtures Act as an Indicator for Species Differentiation?

Theresa Zwickel[1,2*], Sandra M. Kahl[3,4], Michael Rychlik[2] and Marina E. H. Müller[3*]

[1] Federal Institute for Risk Assessment (BfR), Berlin, Germany, [2] Chair of Analytical Food Chemistry, Technical University of Munich, Munich, Germany, [3] Leibniz Centre for Agricultural Landscape Research (ZALF), Müncheberg, Germany, [4] University of Potsdam, Institute of Biochemistry und Biology, Potsdam, Germany

***Correspondence:** Marina E. H. Müller; mmueller@zalf.de

Figure-S2: Possible structures from the literature of unknown detected perylene quinone derivatives in our study

References:

Bashyal, B.P., Wellensiek, B.P., Ramakrishnan, R., Faeth, S.H., Ahmad, N., and Leslie Gunatilaka, A.A. (2014). Altertoxins with potent anti-HIV activity from *Alternaria tenuissima* QUE1Se, a fungal endophyte of *Quercus emoryi*. *Biorg. Med. Chem.* 22(21)**,** 6112-6116. doi: 10.1016/j.bmc.2014.08.039.

Podlech, J., Fleck, S.C., Metzler, M., Burck, J., and Ulrich, A.S. (2014). Determination of the absolute configuration of perylene quinone-derived mycotoxins by measurement and calculation of electronic circular dichroism spectra and specific rotations. *Chemistry (Easton)* 20(36)**,** 11463-11470. doi: 10.1002/chem.201402567.

Stack, M.E., and Prival, M.J. (1986). Mutagenicity of the *Alternaria* metabolites altertoxins I, II, and III. *Appl. Environ. Microbiol.* 52(4)**,** 718-722.

Stierle, A.C., Caddlina, J.H., and Strobel, G.A. (1989). Phytotoxins from *Alternaria alternata*, A Pathogen of Spotted Knapweed. *J. Nat. Prod.* 52(1)**,** 42-47. doi: 10.1021/np50061a003.

Wu, W.B., Yue, G.C., Huang, Q.L., Sun, L.L., and Zhang, W. (2014). A new compound from an endophytic fungus *Alternaria tenuissima*. *J. Asian Nat. Prod. Res.* 16(7)**,** 777-782. doi: 10.1080/10286020.2014.896343.

$C_{20}H_{12}O_6$ – EM(ion⁻)m/z=347.0561

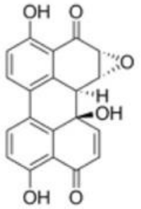

stemphyltoxin III

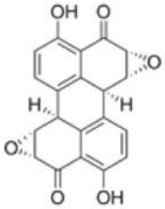

altertoxin III

(Stack and Prival, 1986)

$C_{20}H_{14}O_6$ – EM(ion⁻)m/z=349.0718

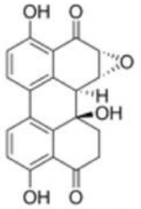

altertoxin II

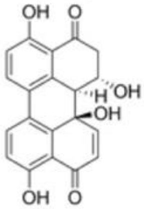

alterperylenol

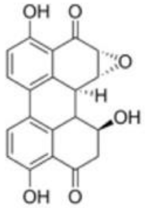

altertoxin V

(Bashyal et al., 2014)

$C_{20}H_{16}O_6$ – EM(ion⁻)m/z=351.0874

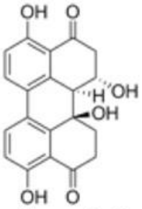

altertoxin I

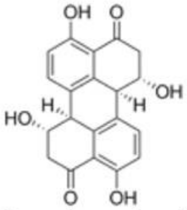

Stemphyperylenol

(Podlech et al., 2014)

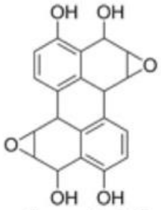

altertoxin IV

(Wu et al., 2014)

$C_{20}H_{14}O_7$ – EM(ion⁻)m/z= 365.0667

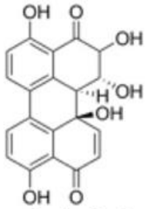

alterlosin I

(Stierle et al., 1989)

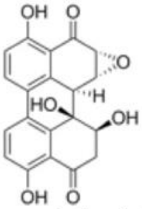

stemphyltoxin I

(Podlech et al., 2014)

$C_{20}H_{16}O_7$ – EM(ion⁻)m/z= 367. 0823

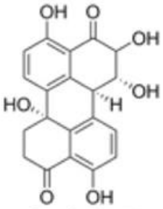

alterlosin II

(Stierle et al., 1989)

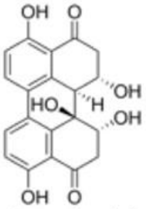

Stemphytriol

(Podlech et al., 2014)

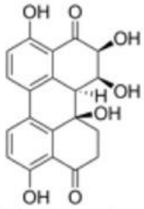

7-epi-8-hydroxy-altertoxin I

(Podlech et al., 2014)

Figure-S2
